# Optical filters made from random metasurfaces using Bayesian optimization

**DOI:** 10.1515/nanoph-2023-0649

**Published:** 2024-01-17

**Authors:** Parker R. Wray, Elijah G. Paul, Harry A. Atwater

**Affiliations:** Department of Electrical Engineering, California Institute of Technology, Pasadena, CA 91125, USA; Thomas J. Watson Laboratories of Applied Physics, California Institute of Technology, Pasadena, CA 91125, USA

**Keywords:** optical filters, random metasurfaces, Bayesian optimization, Mie theory

## Abstract

We theoretically investigate the ability to design optical filters from a single material and a single layer of randomly dispersed resonant dielectric particles, defining a random metasurface. Using a Bayesian and generalized Mie inverse-design approach, we design particle radii distributions that give rise to longpass, shortpass, bandpass, and bandstop spectral bands in the infrared. The optical response is shown to be directly related to electric and magnetic multipole scattering of the constituent particles and their near field coupling. We discuss the effect of the particle size distribution and particle–particle coupling interactions on filter design in random systems lacking long-range order.

## 1 Introduction

Filters designed from disordered metasurfaces may offer a platform to circumvent the Achilles heel of meticulous fabrication. This is because the approach is inherently tolerant to manufacturing error, leading to an increase in throughput and/or a reduced fabrication cost. Furthermore, disordered metasurfaces do not need to rely on multiple materials to achieve a filter response. This benefits applications operating in harsh environments, where issues of different thermal expansion coefficients, chemical stability, miscibility, compliance to mechanical stress, and different resistance to ionizing radiation between materials increases the complexity of filter design [1], [2], [3], [4], [5], [6], [7], [8], [9], [10], [11], [12], [13]. Engineered randomness has a long history of producing emergent phenomena. Historical examples include sub-wavelength scatterers, which catalyzed the field of metamaterials by producing effective media with constitutive parameters not seen in the bulk constituents [14], [15], [16]. Another example is resonant particle absorption and disorder-induced light trapping (e.g., Anderson localization) that has also shown to produce record-breaking broad-band, angle and polarization-invariant near-black-body absorbers. Furthermore, these systems are shown to be scalable and cost effective compared to ordered photonic counterparts [17], [18], [19], [20], [21].

Though the discussion above highlights a case for random metasurfaces, the study of disorder in the field is still an open subject of research. A primary difficulty is that strong light–matter interactions are often necessary in metasurfaces, and this can produce unruly particle–particle coupling effects when the spatial position of particles is not well controlled. It is also important to note that the systems proposed do not fall into the category of effective medium theories. In particular, low harmonic order (electric dipole), non-resonant, and negligible particle–particle coupling are all invalid assumptions in the proposed regime. In fact, it is exactly by leveraging higher harmonics, resonances, and particle–particle coupling that spectral filtering is achieved. A random metasurface therefore requires a robustness to (or appropriate tailoring by) random coupling effects of strongly interacting particles. Otherwise, there would be no emergent collective giving rise to meaningful reflection/transmission bands.

In this manuscript we explore how longpass, shortpass, bandpass, and bandstop spectral features can emerge using only *a single layer* of completely *randomly positioned* and *randomly sized* particles that are *all made from the same material*. This represents an extreme of filter design where multiple material compatibilities and fabrication sensitivity are no longer a primary concern. This also represents a separation in the philosophy of traditional filters. Spectral properties are entirely controlled by probability distributions. For example, instead of optimizing the number of layers, materials, and thickness in a thin film, you optimize a particle distribution and packing fraction in a single layer. The parameters are found using Bayesian inverse design and the results are studied theoretically to pinpoint the governing physics giving rise to the desired spectral response. Our framework, based on generalized multi-particle Mie theory, provides explicit information about the coupling between particles, which is often obscured in inverse design. Furthermore, the optimizer produces fabrication feasible systems that are motivated by well-known massively large-scale and cost effective synthesis and deposition techniques.

The primary goal of the manuscript is two-fold: (1) to present the feasibility of random metasurfaces (e.g., a single layer of randomly distributed particles) in designing optical filters and (2) to present a framework and analysis of the underlying physics giving rise to the filter response in order to motivate future directions and designs.

The first section outlines the theoretical framework and inverse design approach used to derive the transmission, reflection, and absorption from the random metasurface. For brevity, we focus on the main concepts. Detailed derivations are in the Supplementary Information. The second section presents the result of the optimizer, showing the possibility to design the four canonical filter types: longpass, shortpass, bandpass, and bandstop all made from a single layer of particles and of the same material. From this, we outline how the theoretical approach provides insight into the role of the particle shape distribution and the effects of random particle coupling in the final filter response. We conclude with a summary of the results.

## 2 Solving the inverse design problem

The random metasurface problem is formalized as a single layer of randomly shaped scattering elements that are randomly dispersed in the *x*–*y* plane. The film is characterized by a particle shape distribution, $P\left(s\right)$ , and the particles occupy the cross-sectional area filling fraction, *ff*, in the plane. The optimization problem is

(1) $$\begin{array}{c} \underset{\Omega =\left\{P\left(s\right),ff\right\}}{\min}||T_{\text{ideal}}\left(\lambda \right)-T\left(\lambda \right)|{|}^{2}+||R_{\text{ideal}}\left(\lambda \right)-R\left(\lambda \right)|{|}^{2}\\ \text{s.t.},\\ 0\leq P\left(s\right)\leq 1,\\ \int P\left(s\right)ds=1,\\ s_{\min}\leq s\leq s_{\max},\\ 0\leq ff\leq ff_{\max} \end{array}$$

where *T*_ideal_ and *R*_ideal_ are the ideal (user defined) transmission and reflection response, respectively. *ff*_max_ is the upper bound of the particle area filling fraction. *s*_min_ and *s*_max_ are the lower and upper bound of the possible particle radii, respectively. *λ* is the free space wavelength of the incident plane wave excitation. The first two constraints in equation (1) enforce the definition of a probability distribution over particle radii. The third and fourth constraints serve to provide practical bounds on the search space. The minimum and maximum possible particle radii is set based on the particle size parameter, *ks* = (2*π*/*λ*)*s*, which nominally determines the set of possible modes supported in a particle. *s*_min_ and *s*_max_ are constrained such that 0.1 ≤ *ks* ≤ 3. The maximum allowed filling fraction is *ff*_max_ = 60 %, since higher filling fractions approach lattice packing, and we are concerned with random spatial distributions that do not exhibit long-range order. Otherwise, the only other constraint is that particles cannot overlap and must remain within a single layer. I.e., particles do not sit on top, above, or below of one another and cannot fuse together. Particles can (and often do) touch side to side and particle–particle coupling effects can be significant.

Calculating the total transmission, *T*, reflection, *R*, and absorption, *A*, response of the random metasurface relies on three cornerstones that are expanded upon in the Supplementary Information. First, the scattered field formalism is used to explicitly describe how nanoscopic interactions construct emergent macroscopic (film-level) behavior. Second, each particle in the film is expanded into a generalized Mie basis. This allows the effect of particle shape and particle–particle coupling to be represented as a tangible set of atom-like interactions. The third cornerstone is to use Monte Carlo integration to solve the statistical nature of the more general infinite random film problem.

In matrix notation, the Mie expansion of an arbitrary electric field is ***E*** = **Ψ** *c*, where $\Psi \in C^{3\times \left|l\right|}$ is a complex-valued matrix of Mie harmonics and $c\in C^{\left|l\right|}$ is a vector of the basis (scattering) coefficients. *l* = *t*, *n*, *m*, *p* is a unique index defined by the harmonic’s quantum polar, $n\in Z^{+}$ , and azimuthal number, $m\in \left[0,n\right]\cup Z^{+}$ , as well as the harmonic type, *t* (0 = electric-type, 1 = magnetic-type), and parity, *p* (0 = even, 1 = odd). Correspondingly, $\left|l\right|$ is the size of the dimension of all possible harmonic orders necessary to describe the electromagnetic field. Using this method, the governing interaction equation for an arbitrary particle, *a*, embedded in a film of J particles is

(2) $$c_{a}-T_{a}\left(s\right)\overset{J}{\underset{b\neq a}{\sum }}H^{ab}\left(d_{ab}\right)c_{b}=T_{a}\left(s\right)J^{a0}\left(d_{a0}\right)c_{\text{inc}}.$$

$T\in C^{\left|l\right|\times \left|l\right|}$ provides a mapping from the local field a particle experiences to the resulting scattered field the particle emits, $c=Tc_{\text{loc}}$ . The scattered field is a result of the current distribution within the particle that is responding to the external local field. T encapsulates how particle properties such as size, shape, and material define scattering as a response to an arbitrary excitation. $H\in C^{\left|l\right|\times \left|l\right|}$ is a translation operator that describes how a scattered field from particle *b* contributes to the local field onto particle *a*. $J\in C^{\left|l\right|\times \left|l\right|}$ is a similar operator translating the incident plane wave from the origin to the location of particle *a*. Both of these operators are a function of the relative vector distance between particles, $d_{ab}\in R^{3}$ , or the particle’s distance to the origin, $d_{a0}\in R^{3}$ . Further details are given in Sections 1–6 of the Supplementary. The exact solution to the *J* – particle coupling problem is found by writing equation (2) for every particle, then solving the system of *J* coupled equations. Repeating this process for different particle configurations constitutes the Monte Carlo scheme.

In principle there is no closed form solution for the infinite random film problem, given an arbitrary joint shape and spatial distribution [22], [23], [24]. Hence, operations on random variables are solved through Monte Carlo. This allows generalized distributions to be studied by realizing them through computer generation. At each iteration of our algorithm, we first generate *N* instances of a random film of nonoverlapping particles for each *s* in $P\left(s\right)$ . This is done using a custom event-driven particle dynamics algorithm that packs particles to the specified fill fraction, and then moves them randomly to remove artificial correlations as a result of the initial packing. The algorithm is designed to mimic the random motion of uncharged hard particles in a Langmuir–Blodgett trough, which is a practical deposition tool to realizing such a film and holds potential as a large-scale deposition technique [25], [26]. Each iteration of the optimizer involves *S* × *N* × Λ generalized Mie simulations. Here, *S* is the discretization size of the shape distribution. *N* is the number of sampled unique local fields a particle experiences. I.e., for each sampled particle with shape *s*, there are *N* realizations of a unique photonic environment of neighboring particles. Λ is the total number of wavelengths considered. Simulations are in frequency-domain. The generalized Mie simulations are performed using a custom-built code, derived from SMUTHI [27], for increased computation speed. It is also important to note that the method is complete and converges to an exact solution as the multipole order and the number and size of Monte Carlo samples increases. In particular, this approach captures the reflection and transmission from both the coherent and incoherent field. The latter is generally substantially harder to describe analytically and cannot be described by an effective medium theory.

By recasting the random film problem to leverage the orthogonality of the Mie functions, it is possible to substantially increase the speed of electromagnetic calculations. For the optimizer, transmission, reflection, and absorption are defined as

(3) $$\begin{array}{c} T\left(\lambda \right)=1-R\left(\lambda \right)-A\left(\lambda \right)\\ R\left(\lambda \right)=\frac{ff\left\langle \sigma_{\text{sca}}\left(\lambda ,r,s\right)\right\rangle }{1+FBR_{A}}\\ A\left(\lambda \right)=ff\left\langle \sigma_{\text{abs}}\left(\lambda ,r,s\right)\right\rangle . \end{array}$$

⟨⟩ is the expectation operator over the joint particle shape and position distribution. $\left\langle \sigma_{\text{sca}}\left(\lambda ,r,s\right)\right\rangle$ and $\left\langle \sigma_{\text{abs}}\left(\lambda ,r,s\right)\right\rangle$ are the expected scattering and absorption efficiency of a particle within the film. ${\text{FBR}}_{A}\left(\lambda \right)\approx \frac{\left\langle \sigma_{\text{sca}}\left(\theta =0{}^{\circ},\lambda ,r,s\right)\right\rangle }{\left\langle \sigma_{\text{sca}}\left(\theta =90{}^{\circ},\lambda ,r,s\right)\right\rangle }$ is the ratio of the expected scattering in the forward, *θ* = 0°, and backward, *θ* = 90°, directions. This ratio leverages the degeneracy of the Mie harmonics at the poles. The advantage of equation (3) is computational speed. In particular, the FBR_*A*_ reduces two lengthy hemispherical integrations over *O*(*l*!) different multipole permutations to a calculation of $O\left(n^{2}\right)$ that does not require a single numerical integration [28]. This change provides a considerable reduction in computational effort. The power balance relation for the random film is $\left\langle \sigma_{\text{ext}}\right\rangle =\left\langle \sigma_{\text{abs}}\right\rangle +\left\langle \sigma_{\text{sca}}\right\rangle$ , where $\left\langle \sigma_{\text{ext}}\right\rangle$ is the measure of the power removed from the incident plane wave as a result of interference with the collective scattered field emanating from the random metasurface. Unlike isolated particle Mie theory, it is necessary to account for the interference between each particle’s scattered field. This is encapsulated in the scattering efficiency for each particle, *σ*_sca_ = *σ*_sca−i_ + *σ*_sca−*d*_. *σ*_sca−*i*_ ∝ *c* ^†^ *c* is a measure of the power scattered by each particle and $\sigma_{\text{sca }-\text{d }}\propto c_{a}^{†}\sum H^{ab}c_{b}$ accounts for the interference between scattered fields. † denotes the conjugate transpose operation. For historical reasons, *σ*_sca−i_ and *σ*_sca−d_ are termed the “independent” and “dependent” scattering efficiency. Further detail on deriving the random film system can be found in Section 7.

The key insight of equations (1)–(3), is that the film’s total reflection, transmission, and absorption is governed by the ensemble average of the directional scattering and the absorption efficiencies of the individual particles making up the film. These expected values are controlled by the particle shape, *P*(*s*), and spatial position distribution, *P*(***r***). The former nominally dictates the scattering modes a particle will support and is primarily controlled through T . The latter defines the effect of spatial correlation in particle–particle coupling dynamics, which it primarily controlled through H . Note that these two distributions are not uncorrelated as the shape distribution limits the possible particle spatial configurations since particles cannot overlap.

**Figure 1:**
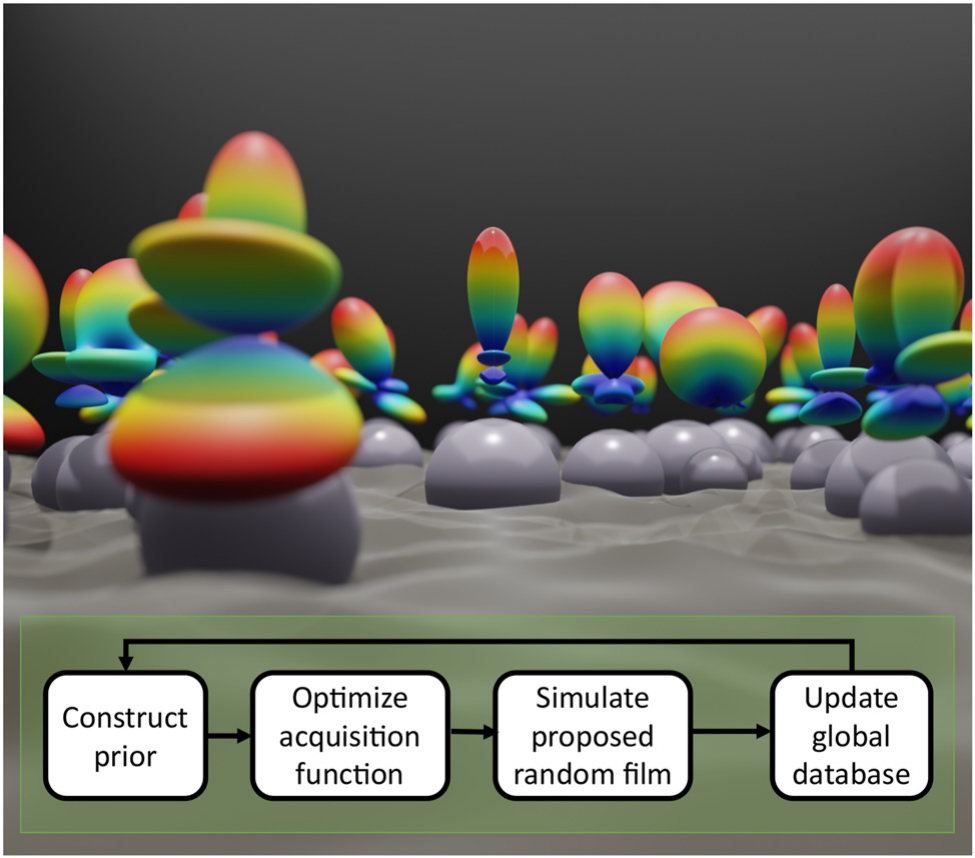
Schematic showing randomly positioned and randomly sized spheres having different far field scattering distribution functions (imaged above each sphere) as a result of near field coupling (the wave function the spheres are immersed in). A flow diagram of the Bayesian optimizer is also shown.

Even with the computational benefits inherent to generalized Mie theory and equation (3), this method poses a computational challenge due to the many simulations necessary to reach convergence in both Monte Carlo and in optimization. To accelerate performance the Monte Carlo process (both the event-driven particle dynamics and electromagnetic calculation) is massively parallelized through a distributed programming scheme using Dask [29]. A graphical image of nanoparticle coupling giving rise to different far field scattering distributions of each particle and the optimization pipeline is shown in Figure 1. All computationally heavy calculations are written in *C* to maximize computation speed.

Besides analytic, algorithmic, and parallelization optimizations of the electromagnetic solver, it is also critical to minimize the number of function calls necessary for the optimizer to reach a satisfying filter performance. Bayesian optimization is a well-suited solution for this problem and the framework we adopt.

Bayesian optimization is a global optimization technique that can minimize the number of evaluations of costly nondifferentiable and noisy objective functions with mixed constraints at moderate dimensions [30]. We use Bayesian optimization based on BoTorch [31] with a Gaussian process prior and the expected improvement acquisition function. This combination gives cheap-to-evaluate surrogates, an analytic form of the acquisition function, and inherently provides a tradeoff between exploitation and exploration of the parameter space [30]. To enable a more efficient reuse of samples, the reflection and transmission curve of each evaluated filter is saved in a global dataset and the next sample is based on the totality of the shared data. This is because $P\left(s\right)$ and *ff* uniquely define the filter, which, for example, cannot simultaneously be a good shortpass and longpass filter. Therefore, for example, the results from the longpass filter optimization helps increase the information available to the Bayesian prior for the shortpass optimizer. Each optimization utilizes its own objective but shares information about the evaluation points of all other running and past run results. Convergence of the optimization process is shown in the Supplementary Information.

## 3 Filters made from a random metasurface

**Figure 2:**
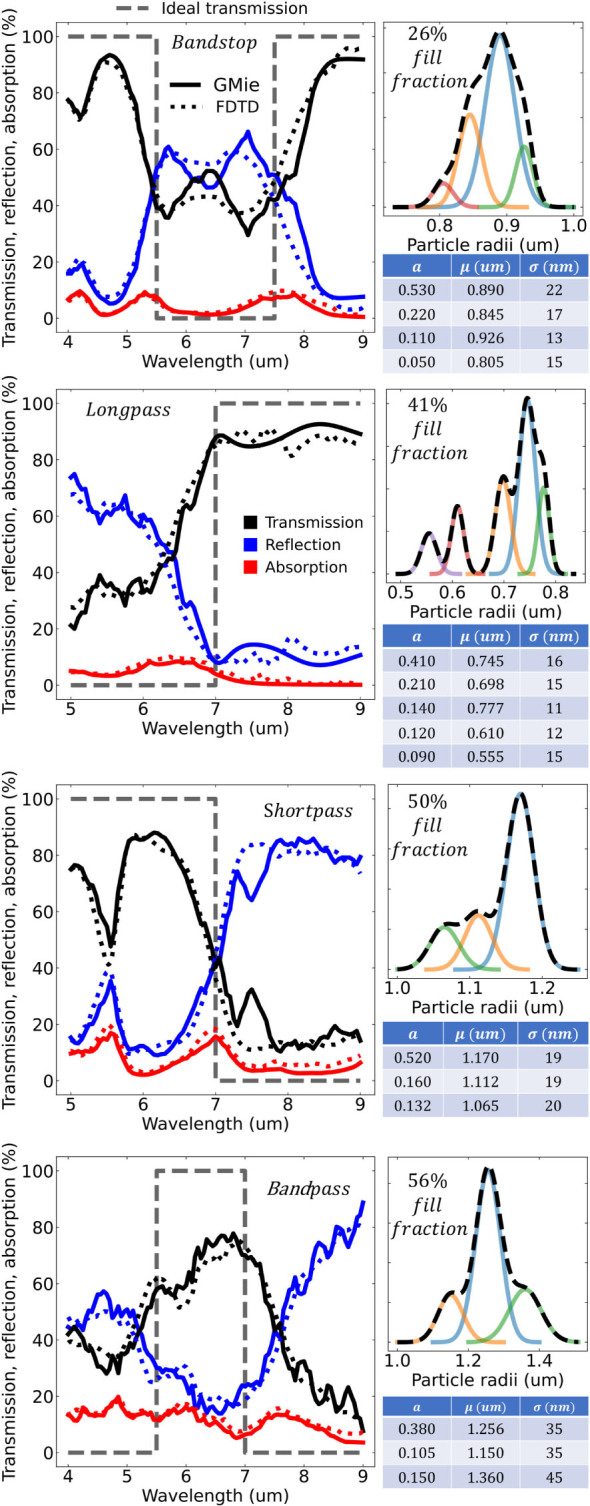
Transmission (black), reflection (blue), and absorption (red) response for the four particle filters. Solid lines are calculated using the generalized Mie method. Dotted lines are calculated using finite-difference time-domain (FDTD). The ideal transmission is in dashed shaded black. For each filter, the upper right figure is the film’s particle size probability distribution and area fill fraction. The size distribution is decomposed into Gaussian distributions and the table for the Gaussian amplitudes (*a*), mean value (*μ*), and standard deviation (*σ*) are shown in the corresponding table below the figure.

Figure 2 shows the resulting design of bandstop, longpass, shortpass, and bandpass spectral features in the infrared, using the approach outlined in Section 2. Overall, low absorption loss filters can be achieved with stopbands ranging from 50 % to over 90 % and passbands from 40 % to over 80 %. In all cases, our result is compared to full-wave finite-difference time-domain (FDTD, dotted lines) simulations to highlight the accuracy of our framework. Both methods show excellent agreement, further validating the use of this method to property represent complex interparticle coupling dynamics in random systems.

The filters are made completely out of a single layer of randomly placed germanium particles with optimized radii distributions (black dashed overlay line), and unique packing fractions, shown in the top right. In all cases the optimal radii distributions can be constructed from the sum of simple Gaussian distributions (colored solid lines). This supports the feasibility of designing such filters in experiment. The Gaussian distribution is the common default distribution found in many particle synthesis and size-filtering techniques [32], [33], [34], [35], so one can simply mix different batches of synthesized particles at the proper weight fraction to produce the optimal distribution.

The spectral range was chosen so that the refractive index of germanium is approximately constant (*η* = 4.17 + *i*5 × 10^−3^). By choosing a region of high dielectric index and low material loss we show that each filter’s stopband is not a result of absorption, but instead a result of strong multiple scattering and interference effects. This is a fundamentally different approach compared to the small particle systems that are well described by effective media. For example, Supplementary Section 9 gives a comparison to the Maxwell–Garnett and Bruggeman mixing formulas, which show poor modeling performance. The approach is also a fundamentally different compared to Mie resonant systems reliant on periodicity. For example, Supplementary Section 10 gives a comparison to a packed periodic square lattice.

**Figure 3:**
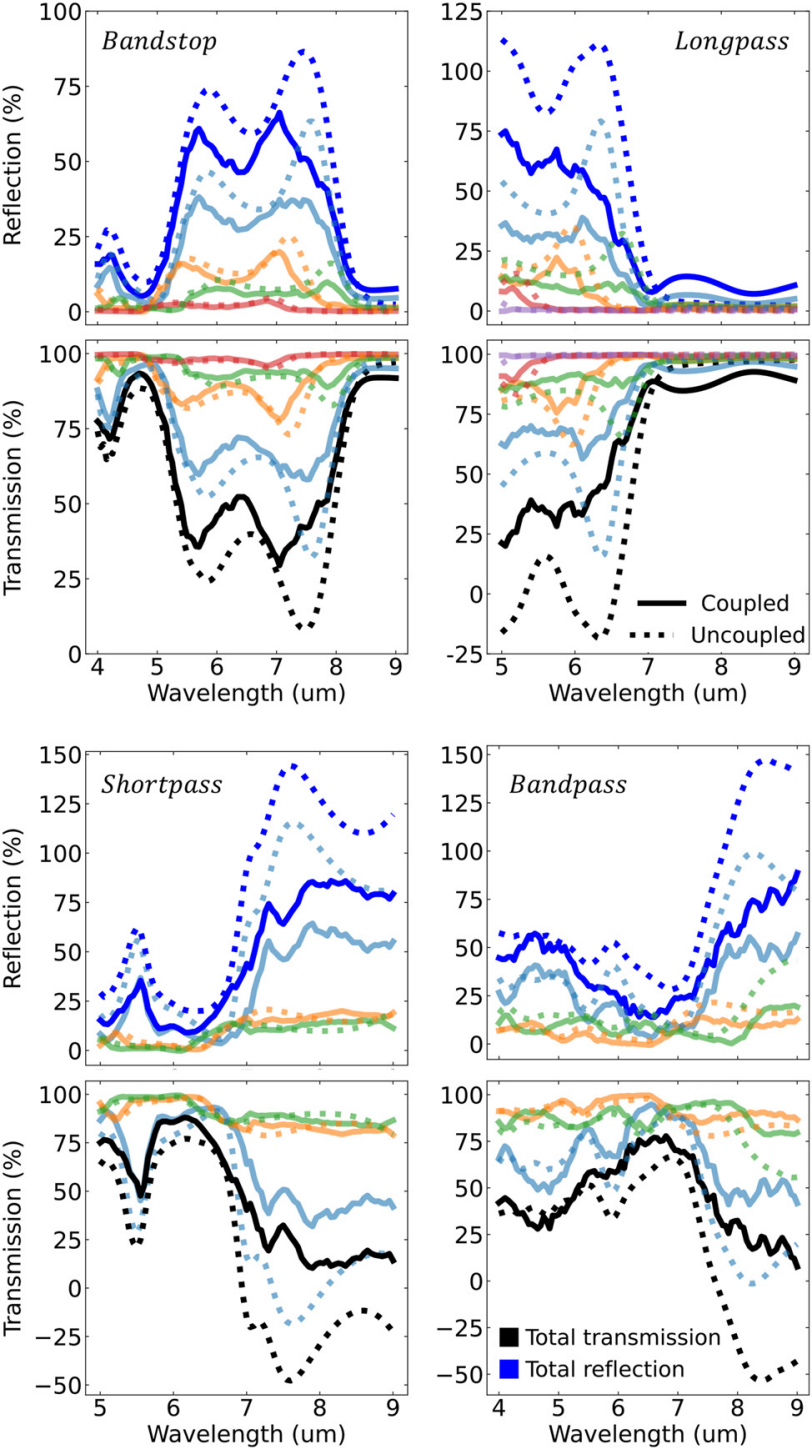
Reflection (blue) and transmission (black) response of each particle filter decomposed to resolve the contributions from each Gaussian distribution making up the particle film. The Gaussian-resolved reflection and transmission are color coded according to the colors of the Gaussian fit in Figure 2. Solid line curves are calculated using generalized Mie theory accounting for particle coupling described by equation (4). Dotted line curves are calculated assuming no particle coupling.

Since the framework utilizes the scattered field formalism, it is possible to decompose the filter response based on particle size in order to study the effect of the particle size distribution. Clearly, such an analysis is not possible in full-wave techniques that only record the total field. Figure 3 decomposes each filter’s reflection and transmission spectra based on the underlying Gaussian size distributions in Figure 2. Figure 3 shows that the primary (largest, light blue) Gaussian is also the primary contribution to the overall filter response. This is sensible as our analytic derivation in the supplementary shows the filter response is linearly proportional to the shape distribution. The remaining Gaussian distributions, clustering close to the primary distribution, then act as higher order correction terms chosen by the optimizer to broaden and flatten pass/stopbands.

Figure 3 also compares the filter response if particle–particle coupling effects were removed (dashed lines). The purpose of comparing to a non-physical scenario of filters made from noninteracting particles is to contrast how coupling alters the overall spectral response. This also highlights a benefit to the theoretical construction. Simulations assuming no particle coupling can easily be derived by eliminating the particle coupling operator, H=0 , between particle pairs. Therefore, you can “turn on” and “turn off” particle coupling effects at will by including or removing the H term, respectively. In Figure 3, the uncoupled system produces a non-physical total reflection and transmission spectra. This is expected as the H=0 assumption does not define a proper power balance relationship. The inaccuracy is best seen near particle resonances, where the electrical cross sections of individual particles are more likely to overlap. Recall, that physical particles cannot overlap. But nothing prevents electrical (e.g., scattering and absorption) cross sections from overlapping. When coupling is not accounted for in the bookkeeping, then it is possible that the sum of all electrical cross sections from all particles becomes larger than the extent of the *x*–*y* plane. Clearly this is nonphysical as the extinction theorem outlines that the total particle system cannot extinguish more power than what is supplied by the incident plane wave. Correspondingly, these nonphysical regions in the uncoupled spectra highlight where net quenching must occur in order to maintain power balance. The quenching is a direct result of particle–particle coupling effects. Further detail on the role of particle coupling versus particle size can be found in Section 8 of the Supplementary.

Strictly speaking, the uncoupled assumption is accurate only in the limit of vanishing electrical cross sections and/or vanishing fill fraction. This is a common stipulation in effective medium theories that clearly does not apply in our case. With that said, even though the uncoupled predictions are not physical, they still predict well the spectral location of stopbands and passbands. This may provide valuable insight to future work in random metasurface optimization. First, uncoupled calculations are exceptionally faster and more memory efficient to calculate compared to coupled calculations. This is because particle coupling forms a large set of coupled linear equations that must be constructed and then solved. In future works, the optimizer could first use the uncoupled model to quickly rule out areas of the search space that clearly do not match the objective function. Bayesian optimization provides a clear theoretical interpretation of such a low-fidelity simulation as adding additional information to the Bayesian prior. Furthermore, the nonphysical regions in the uncoupled spectra could be penalized, weighted, or smoothed to mimic the necessary net reduction in particle cross sections. This then gives a low fidelity surrogate model for coupling effects.

**Figure 4:**
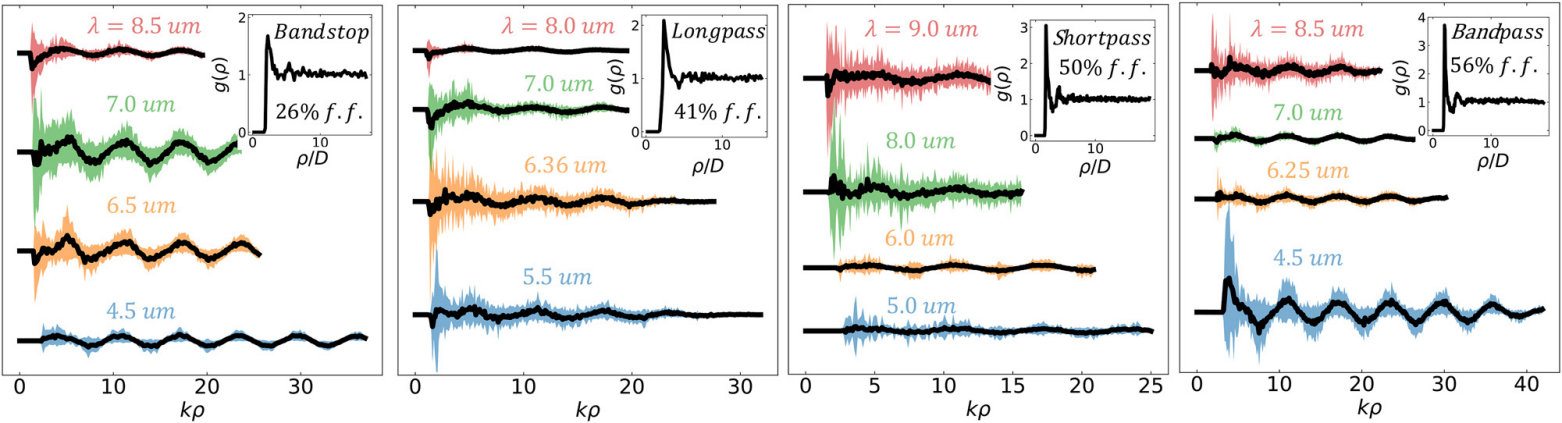
The statistics of *σ*_sca−d_ as a function of radial distance, *ρ*. Black lines are average values of *σ*_sca−d_ and the shaded region gives the standard deviation. Two representative spectral locations are shown in both the passband and the stopband of each filter. Radial distances are normalized by the wavenumber, *k* = 2*π*/*λ*. The upper right quadrant of each figure plots the particle–particle pair correlation function for each filter. In this plot radial distances are normalized by particle diameter, *D*.

Uncoupled simulations correctly predicting the location of passbands and stopbands implies a dominance in $\left\langle \sigma_{\text{sca}-\text{i}}\right\rangle$ over $\left\langle \sigma_{\text{sca}-\text{d}}\right\rangle$ in defining the spectral shape. This is sensible when the structure factor of the film lacks a strong coherence effect. Then T can dominate over the role of H . With that said, though the structure factor is not strongly coherent, particle coupling still plays an important role to maintain global power balance. To study how particle coupling is dependent on the film’s structure factor, Figure 4 plots the statistics of *σ*_sca−d_ as a function of the radial distance, *ρ*, between particles. Recall from Section 2 that *σ*_sca−d_ is the portion of a particle’s scattering efficiency accounting for the interference with other scattered fields. The solid black lines in Figure 4 plot the expectation of the dependent scattering efficiency, $\left\langle \sigma_{\text{sca}-\text{d}}\right\rangle$ . The shaded region gives the standard deviation. The statistics of *σ*_sca−d_ are shown at four representative spectral locations. Two locations are in the passband and two are in the stopband of each filter. In Figure 4, the particle–particle pair correlation function, *g*(*ρ*), for each filter is also shown in the upper right of each plot. This gives reference to the spatial structure factor of the film. In all cases, the radial distribution function resembles the Percus–Yevick equation for hard spheres. There is clear short-range order and correlation increases with increasing filling fraction. No long-range order exists. At large interparticle distances (*kρ* ≥ 10), $\left\langle \sigma_{\text{sca}-\text{d}}\right\rangle$ decays with a spherical Bessel-like oscillation. This indicates uncorrelated interactions consistent with a lack of long-range order.

In almost all cases $\left\langle \sigma_{\text{sca}-\text{d}}\right\rangle$ has a net deleterious effect at short-range. This evident by the dip near the minimal distance and is consistent with the idea that particles reduce their neighbor’s interaction cross section in order to maintain power balance. For an individual particle, the local photonic environment can strongly vary. This is evident by the large standard deviations that are particularly strong at short distances. There is certainly strong nearest neighbor and near field coupling occurring in these systems. In all cases, when the particle is approaching a scattering peak, the nearest neighbor coupling interactions are stronger and more varying. This supports the geometric interpretation discussed in Figure 3 that coupling is a result of scattering cross sections having a greater degree of mutual overlap.

**Figure 5:**
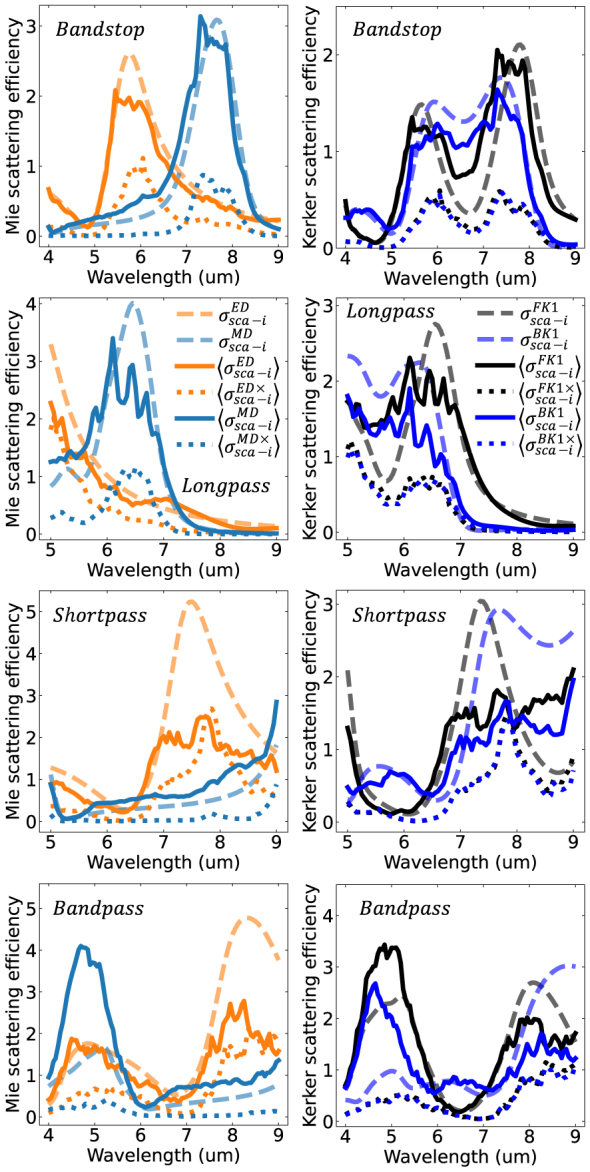
Independent scattering efficiency of all four designed filters. The left column shows the contribution of the electric (orange) and magnetic (light blue) dipole harmonics to the scattering efficiency. The first order forward (black) and backward (blue) Kerker representation is shown in the corresponding figure to the right. In all cases solid lines denote to the independent efficiency arising from harmonics with parity aligned with the expected orientation according to the plane wave polarization. E.g., given a linearly polarized incident plane wave, the expected orientation is electric dipoles aligned with the incident electric field and magnetic dipoles are aligned with the incident magnetic field. Dotted lines are the contributions of harmonics excited in the cross-polarization orientation. E.g., electric dipoles aligned with the incident magnetic field and magnetic dipoles aligned with the incident electric field. Dashed lines plot the independent scattering efficiency assuming no multiple scattering contribution to the local field. I.e., uncoupled particles. Since the cross-polarization term is a direct result of the multiply scattered field, the uncoupled system has no cross-polarization harmonics. For readability, the legend uses $\left\langle \cdot \right\rangle \left(=\left\langle \left\langle \cdot \right\rangle |s\right\rangle \right)$ to denote film-level efficiencies that incorporate particle coupling. This allows unbracketed terms to denote uncoupled film-level efficiencies.

To study the role atom-like electric and magnetic-type Mie harmonics play in defining the filter response and coupling effects, Figure 5 plots the average independent scattering efficiency, $\left\langle \sigma_{\text{sca}-\text{i}}\right\rangle$ decomposed by Mie harmonic (left column). Recall, from Section 2 that *σ*_sca−i_ ∝ *c* ^†^ *c* defines the scattered power emanating directly from individual particles within the film. The right column of Figure 5 decomposes *σ*_sca−i_ in terms of Kerker harmonics. This provides additional insight into the degree of directional scattering asymmetry. This will be discussed in detail later. Figure 5 also plots the result from filters made of uncoupled particles (dashed lines). The left column of Figure 5 plots the electric and magnetic dipole scattering efficiency. For uncoupled particles, only harmonics with the *m* = 1 azimuthal number have nonzero scattering coefficients. In a coupled random film, all integer values of *m* ∈ [0, *n*] can be populated. Therefore, sums are performed over *m* since we are not concerned with resolving azimuthal variations. Particle coupling also induces electric and magnetic-type multipoles of opposite parity to those expected by the polarization state of the external plane wave excitation. Therefore, cross-polarized scattering is another feature not seen in the uncoupled approximation (or in planar-film filters). To clarify, the cross polarized terms have a × superscript in the legend and are represented by dotted lines.

For all filters, the electric and magnetic-type dipoles are the primary harmonics driving the filter response. Strong reflections are then a result of strong backward scattering near the middle electric and magnetic dipole crossing point. This is analogous to the backward Kerker effect for random film systems [28]. This will be further discussed when presenting the Kerker transform later. The contribution of higher order harmonics is presented in the Supplementary Information.

On average the coupled system’s scattering harmonics have the same spectral shape and location compared to uncoupled predictions. This again substantiates the result of Figure 3 and indicates the role of T in defining the scattering spectra for these systems. Resonant peaks are reduced as a result of interparticle coupling. The reduction of resonant peaks is clearly more pronounced when particles are packed at a higher filling fraction. In multiple cases the electric dipole resonance is shown to be more affected by interparticle coupling compared to the magnetic dipole resonance. This is sensible as the latter comes from a strong closed loop oscillation of weakly damped bound charges, which can be seen deep into the core of the particle. In contrast, the charge distribution of the electric dipole is distributed on the outer edges of the particle, making it more susceptible to changes by the external environment. In the shortpass and bandpass filter, the electric dipole resonance is reduced by more than half, lacks a well-defined peak, and the cross polarized harmonics are of near equal magnitude to the same-polarized harmonics. Such deviation of the scattering behavior compared to an uncoupled particle brings the question: If the harmonic profile dictates the direction of scattered photons and the shortpass and bandpass coupled system are strongly different to their uncoupled analogy, why does Figure 3 show they have similar spectral behavior?

Though the Mie harmonics provide insight into the difference in the robustness of electric and magnetic-type resonances as a result of particle coupling, these harmonics do not provide insight into the directionality of scattering. For sufficiently high-index and low-loss dielectrics, in the Mie size regime, directional scattering can be found at crossing points of the magnetic and electric harmonics. This effect, termed the Kerker effect, is strongly dependent on both the relative amplitude and phase of the interfering harmonics. Furthermore, cross polarization terms must also be considered in random systems [28]. Strictly speaking, directional scattering is the result of coupling between *O*(*tnmp*!) harmonic pairs because the orthogonality conditions of the Mie harmonics cannot be leveraged on the hemisphere. Furthermore, the analysis of both amplitude and phase is necessary as directionality is a coherent interference phenomenon.

The Kerker harmonics are a basis of highly directional forward and backward-type multipoles that are designed to better elucidate features related to the directionality of scattering. These harmonics can be constructed from a linear transform of the outward propagating (Hankel) Mie harmonics as,

(4) $$\begin{array}{cc} \Upsilon_{\text{nmp}}^{f}\left(r\right) & ={\left(i\right)}^{n}\left(\Psi_{\text{nmp}}^{M}\left(r\right)+{\left(-1\right)}^{p}i\Psi_{\text{nm}1\;-\text{ p}}^{E}\left(r\right)\right)\\ \Upsilon_{\text{nmp}}^{b}\left(r\right) & ={\left(-1\right)}^{\left(n+m+1\right)}{\left(i\right)}^{n}\left(\Psi_{\text{nmp}}^{M}\left(r\right)\right.\\ & \;\left.+{\left(-1\right)}^{1-p}i\Psi_{\text{nm}1\;-\text{ p}}^{E}\left(r\right)\right), \end{array}$$

where *f* and *b* denote the basis of forward and backward-type directional Kerker harmonic, ***Υ***, respectively. *E* and *M* denote electric and magnetic-type Mie harmonic, ***Ψ***, respectively. Correspondingly, the Kerker scattering coefficient, *d*, is related to the Mie coefficient, *c*, through the transform

(5) $$\begin{array}{ll} d_{\text{nmp}}^{t} & =\frac{1}{2}{\left(-1\right)}^{t\left(n-m-1\right)}{\left(-i\right)}^{n}\\ & \;\times \left(c_{\text{nmp}}^{M}+{\left(-1\right)}^{1-t-p}ic_{\text{nm}1\;-\text{ p}}^{E}\right) \end{array}$$

where again $t\in \left\{f=0,b=1\right\}$ denotes a forward or backward-type, respectively. Equations (4) and (5) define an element-wise transform where the electric field is then expanded in terms of Kerker harmonics as $E=\Upsilon d\in C^{3}$ .

A primary benefit of the Kerker basis relevant to the current analysis is that directional scattering can be inferred from the forward and backward decomposition of the total scattering efficiency. I.e., $\sigma_{\text{sca}-\text{i}}=\sigma_{\text{sca}-\text{i}}^{f}+\sigma_{\text{sca}-\text{i}}^{b}$ . This leverages the properties of the Kerker harmonics to simplify analysis regarding photon redirection.

The right column of Figure 5 plots the independent scattering efficiency decomposed into Kerker harmonics. Again, the sum over all azimuthal numbers is performed and cross polarization is referenced to the polarization of the external plane wave. Under the Kerker basis, both the first order forward and backward directional harmonic are predominant for all four filters. This is a result of the dominance of the dipole modes in the Mie basis. In all cases, the Kerker backward harmonic shows a strong contribution in each filter’s respective stopband. This is also the middle crossing point between the electric and magnetic dipole mode. In the stopband of the bandstop filter, the backward Kerker harmonic is dominant compared to the forward Kerker harmonic. This indicates preferential backward-dominant scattering in that region. Furthermore, both the bandstop and longpass filter have forward dominant scatting in their long-wavelength passbands.

Though, anomalous directional scatting is present in some filters, it is not the predominant factor in defining each filters performance. In fact, the stopband of the longpass, shortpass, and bandstop filter are all characterized by forward and backward harmonics having approximately the same scattering efficiency. No anomalous directional scattering is occurring in the stopband regions. This strongly contrasts the prediction of uncoupled particles, which attribute all stopbands to highly directional backward-dominant scattering. The question is then, why does the coupled system still have a stopband despite lacking appreciable backward dominated scattering? Furthermore, why does the uncoupled particle approximation still show a similar overall shape in Figure 3, despite predicting strong directional scattering not seen in the coupled counterpart?

They key insight is that the reflection is driven solely by backward particle scattering, which is proportional to $\sigma_{\text{sca}-\text{i}}^{b}$ . Therefore, from the point of view of the optimizer, it is not necessary to simultaneously tune $\sigma_{\text{sca}-\text{i}}^{f}$ as an independent parameter. Instead, it is only necessary to ensure a strong backward harmonic in the stopband and a weak backward harmonic in the passband. The behavior of the forward scattering Kerker harmonic is taken care of by energy conservation. I.e., the extinction terms will suppress scattering in the transmission region when necessary. Therefore, thought the ratio between forward and backward harmonics is not preserved for all filters between the coupled and uncoupled systems, the location of increased/decreased backward scattering is preserved and this is the defining parameter. Thus, both systems predict a similar filter type.

## 4 Conclusions

Using Bayesian optimization, we inversely design particle size probability distributions and packing fractions which give rise to random metasurfaces producing the four fundamental filters (bandpass, shortpass, longpass, and bandstop) in the infrared. These filters are made from a *single material* and a *single monolayer* of randomly distributed Mie resonant particles, where the key design parameters are given by probability distributions. This represents a uniquely different approach to filter design compared to traditional methods such as thin films, ordered metasurfaces, photonic crystals, or effective medium mixtures.

We outline a massively parallelizable Monte Carlo integration technique to solve the total transmission, reflection, and absorption response arising from a monolayer of randomly distributed and arbitrarily shaped particles at packing fractions which lack long range order. This method is based on the generalized Mie technique and fully accounts for multiparticle coupling, including nearfield interactions of highly Mie resonant particles supporting both electric and magnetic-type resonances. The approach shows great agreement with (massively large) random film simulations using finite-difference time-domain using a fraction of the corresponding computational time and resources. The method also provides unique insights into how the collective film response is driven by the statistical properties of the constituent particles within the film. This provides a link between global film response and parameters such as particle size distribution, which can be designed using an appropriate fabrication method. To the best of our knowledge this is the first report to solve a problem of this kind without relying on *a priori* assumptions on the behavior of the local field or full-wave simulations, which lack a strong theoretical framework for analysis.

Given the well-defined theoretical framework, we study the effect of both the particle distribution and packing fraction on individual particle scattering behavior, particle–particle coupling, and how these parameters give rise to the overall filter response. We directly show that the multiply scattered field is appreciable primarily at nearest-neighbor distances where the particle radial distribution function is highly correlated. Particle–particle coupling is shown to primarily cause a reduction in electrical cross sections, on average, in order to maintain power conservation. Though individual particle clusters can vary greatly. The net reduction in electrical cross sections in comparison to isolated particle counterparts is more pronounced when particles are at a scattering resonance and there is more mutual overlap in scattering cross sections. Despite strong interparticle coupling, uncoupled simulations give good predictions to the spectral location of the pass and stopbands. From a harmonic analysis we show that even though these systems predict different behavior on the nanoscopic (individual particle) level, resulting macroscopic behavior is similar enough to warrant use in the optimization procedure as a low-fidelity surrogate and that this approximation can be substantiated by theory.

## Supporting Information

Derivation of our analytic approach for solving the random film problem. Comparison of the particle filters with calculations of isolated particles using traditional Mie theory. Comparison of the particle filters to effective medium and periodic array equivalents. Coordinate system and minimization curves of the optimizer. Harmonic analysis of each particle filter beyond the dipole harmonics.

## Supplementary Material

Supplementary Material Details

## Abbreviations

GMie: Generalized Mie Theory
FDTD: Finite-Difference Time-Domain.
