# Supplementary material for: Optical filters made from random metasurfaces using Bayesian optimization
Source: Nanophotonics. 2024 Jan 17;13(2):183–93. doi: 10.1515/nanoph-2023-0649 (PMC11614330; doi:10.1515/nanoph-2023-0649)
Supplement: Supplementary file 1 — Supplementary Material Details [file j_nanoph-2023-0649_suppl_001.docx]

**Optical filters made from random metasurfaces**

**using Bayesian optimization**

Parker R. Wray^‡^, Elijah Paul^†^, and Harry A. Atwater, ^†^

‡Department of Electrical Engineering, California Institute of Technology, Pasadena, CA 91125, USA

†Thomas J. Watson Laboratories of Applied Physics, California Institute of Technology, Pasadena, CA 91125, USA

1. **Definition of the generalized Mie harmonics**

We start by defining the scalar function

|  |  |
| --- | --- |
| $\psi_{nm}^{\left( \nu\right)}\left( \boldsymbol{r} \right)= \left\{ \begin{matrix} j_{n}\left( kr \right)Y_{nm}\left( \cos\left( \theta\right),\phi\right) & \nu=1, r\neq\infty\\ h_{n}\left( kr \right)Y_{nm}\left( \cos\left( \theta\right),\phi\right) & \nu=3, r\neq0 \end{matrix} \right.$ | S1 |
|  |  |

where $j_{n}\left( kr \right)$ and $h_{n}^{(1)}\left( kr \right)$ are the spherical Bessel (incoming) and Hankel (regular) functions of the first kind, respectively. When $\nu=3$, the spherical basis is based on the spherical Hankel function, which satisfies the Silver-Muller radiation condition and is singular at the origin. This expansion is used to represent scattered/emissive fields. $Y_{nm}\left( \cos\left( \theta\right),\phi\right)$ are the spherical harmonics of quantum polar number, $n$, and quantum azimuthal number, $m$. From the scalar functions we can expand an arbitrary field into a basis of spherical waves as

|  |  |
| --- | --- |
| $\boldsymbol{E}\left( \boldsymbol{r} \right)= \sum_{p=1}^{2} \sum_{n=1}^{N} \sum_{m= -n}^{n} c_{nmp}\boldsymbol{\Psi}_{nmp}^{\left( \nu\right)}(\boldsymbol{r})=\boldsymbol{\Psi}^{\left( \nu\right)}c$, | S2 |
|  |  |

where

|  |  |
| --- | --- |
| $\boldsymbol{\Psi}_{nm1}^{\left( \nu\right)}\left( \boldsymbol{r} \right)=\frac{1}{\sqrt{2n\left( n+1 \right)}}e^{im\phi}z_{n}^{\left( \nu\right)}\left( kr \right)\left( im\pi_{n}^{\left\vert m \right\vert}\left( \cos\theta\right){\hat{\boldsymbol{e}}}_{\theta}-\tau_{n}^{\left\vert m \right\vert}\left( \cos\theta\right){\hat{\boldsymbol{e}}}_{\phi} \right)$ | S3 |
|  |  |

and

|  |  |
| --- | --- |
| $\boldsymbol{\Psi}_{nm2}^{\left( \nu\right)}\left( \boldsymbol{r} \right)=\frac{1}{\sqrt{2n\left( n+1 \right)}}e^{im\phi}\frac{1}{kr}\left( \begin{aligned} n\left( n+1 \right)z_{n}^{\left( \nu\right)}\left( kr \right)P_{n}^{\left\vert m \right\vert}\left( \cos\theta\right){\hat{\boldsymbol{e}}}_{r} \\ +\partial_{kr}\left( krz_{n}^{\left( \nu\right)}\left( kr \right) \right)\left( \tau_{n}^{\left\vert m \right\vert}\left( \cos\theta\right){\hat{\boldsymbol{e}}}_{\theta}+im\pi_{n}^{\left\vert m \right\vert}\left( \cos\theta\right){\hat{\boldsymbol{e}}}_{\phi} \right) \end{aligned} \right)$*.* | S4 |
|  |  |

Also $\tau_{n}=\frac{dP_{n}^{1}(\cos\left( \theta\right))}{d\theta}$ and $\pi_{n}=\frac{1}{sin\theta}P_{n}^{|m|}(\cos\left( \theta\right))$ are the angle-dependent basis functions. $P_{n}^{m}\left( \cos\left( \theta\right) \right)$ is the associated Legendre polynomial with the argument of polar angle, $\theta$. The first equality in S2 is written explicitly in summation notation. The second equality in equation S2 assumes the compact matrix form used in the main text, where $\boldsymbol{\Psi}^{\left( \nu\right)}\in\mathbb{C}^{3\times l}$ and $c\in\mathbb{C}^{l}$. This form is denoted by the lack of an explicit $nmp (=l)$ subscript.

1. **Scattering response operator of the sphere**

Due to the rotational symmetry of the sphere, $\mathbb{T}$ is nonzero only on the diagonals. At those locations the values are given by the Mie scattering solution of the sphere. The coefficients are $a_{n}$ for transverse magnetic (TM) modes and $b_{n}$ for transverse electric (TE) modes. The coefficients are found by applying the boundary conditions of field continuity between the particle and environment, leading to the well-known Mie solutions for non-magnetic particles

|  |  |
| --- | --- |
| $a_{n}=\frac{\eta\psi_{n}\left( \eta x \right)\psi_{n}^{'}\left( x \right)-\psi_{n}\left( x \right)\psi_{n}^{'}\left( \eta x \right)}{\eta\psi_{n}\left( \eta x \right)\xi_{n}^{'}\left( x \right)-\xi_{n}\left( x \right)\psi_{n}^{'}\left( \eta x \right)}$*,* | S5 |
|  |  |

and

|  |  |
| --- | --- |
| $b_{n}=\frac{\psi_{n}\left( \eta x \right)\psi_{n}^{'}\left( x \right)-\eta\psi_{n}\left( x \right)\psi_{n}^{'}\left( \eta x \right)}{\psi_{n}\left( \eta x \right)\xi_{n}^{'}\left( x \right)-\eta\xi_{n}\left( x \right)\psi_{n}^{'}\left( \eta x \right)}$*,* | S6 |
|  |  |

where $\psi_{n}(\rho)=\rho j_{n}(\rho)$ and $\xi_{n}\left( \rho\right)=\rho h_{n}^{\left( 1 \right)}(\rho)$  [1]. The prime superscript denotes differentiation. The argument $\rho=\frac{2\pi r}{\lambda_{0}}$ is the size parameter from the main text. $\eta=n_{s}/n_{e}$ is the ratio of the complex refractive index of the particle ($n_{s}$) and the host environment ($n_{e}$).

1. **Definition of the generalized Mie vector harmonic translation operators**

The translation operator $\mathbb{H}^{ab^{T}}(d)$ is defined as,

|  |  |
| --- | --- |
| $\boldsymbol{\Psi}_{l}^{\left( 3 \right)}\left( \boldsymbol{r}-\boldsymbol{r}_{j}+\boldsymbol{d}_{\boldsymbol{ab}} \right)= \sum_{l^{'}} \mathbb{H}_{ll^{'}}(\boldsymbol{d}_{ab}\boldsymbol{)} \boldsymbol{\Psi}_{l^{'}}^{\left( 1 \right)}\left( \boldsymbol{r}-\boldsymbol{r}_{j} \right)$ = $\mathbb{H}^{ab^{T}}\left( {\tilde{\boldsymbol{d}}}_{ab} \right)\boldsymbol{\Psi}^{\left( 1 \right)}\left( \boldsymbol{r}-\boldsymbol{r}_{j} \right)$, | S7 |
|  |  |

where, $T$ denotes the transpose and, again, $l=mnp$ is a compact notation denoting the sum over all polar numbers and parity. The necessity to define the transpose of the translation operator will become apparent in the fourth section, when defining the fundamental interaction equation. The meaning of operator is to expand a scattered/emissive field from particle a, which is originally represented in a basis of outgoing harmonics at location $\boldsymbol{r}_{a}$, into an incoming expansion at location $\boldsymbol{r}_{b}$. This operator is solely a function of the distance between the two locations, $\boldsymbol{d}$. Each element of the operator is calculated as

|  |  |
| --- | --- |
| $\mathbb{H}_{ll^{'}}=\delta_{pp^{'}}\mathbb{A}_{mn,m^{'}n^{'}}+(1-\delta_{pp^{'}})\mathbb{B}_{mn,m^{'}n^{'}}$, | S8 |
|  |  |

where

|  |  |
| --- | --- |
| $\mathbb{A}_{mn,m^{'}n^{'}}=e^{i\left( m-m^{'} \right)\phi_{d}} \sum_{y=\vert n-n^{'}\vert}^{n+n^{'}} a_{5}(n,m\left\vert n^{'},m^{'} \right\vert y)h_{y}^{(1)}\left( kd \right)P_{y}^{\vert m-m^{'}\vert}\left( \cos\left( \theta_{d} \right) \right)$ | S9 |
|  |  |

and

|  |  |
| --- | --- |
| $\mathbb{B}_{mn,m^{'}n^{'}}=e^{i\left( m-m^{'} \right)\phi_{d}} \sum_{y=\vert n-n^{'}\vert}^{n+n^{'}} b_{5}(n,m\left\vert n^{'},m^{'} \right\vert y)h_{y}^{(1)}\left( kd \right)P_{y}^{\vert m-m^{'}\vert}\left( \cos\left( \theta_{d} \right) \right)$. | S10 |
|  |  |

The relation $\mathbb{H}^{ab^{T}}=\mathbb{J}^{ab^{T}}+i\mathbb{Y}^{ab^{T}}$ is now explicitly shown to be a result of equation S9 and S10, where $h_{y}^{(1)}=j_{y}+iy_{y}$. Therefore, $\mathbb{J}^{ab^{T}}$ is simply the same formula defining $\mathbb{H}^{ab^{T}}$, but now using the spherical Bessel function of the first kind instead of the spherical Hankel function of the first king, in equations S9 and S10. Finally, the transition functions are defined as

|  |  |
| --- | --- |
| $a_{5}\left( n,m\left\vert n^{'},m^{'} \right\vert y \right)=i^{\left\vert m-m^{'} \right\vert-\left\vert m \right\vert-\left\vert m^{'} \right\vert+n^{'}-n+y}\left( -1 \right)^{m-m^{'}}\sqrt{\frac{\left( 2n+1 \right)\left( 2n^{'}+1 \right)}{2n\left( n+1 \right)n^{'}\left( n^{'}+1 \right)}}$  $\left( n\left( n+1 \right)+n^{'}\left( n^{'}+1 \right)-y\left( y+1 \right) \right)\sqrt{2y+1}\times\left( \begin{matrix} n & n^{'} & y \\ m & -m^{'} & -(m-m^{'}) \end{matrix} \right)\left( \begin{matrix} n & n^{'} & y \\ 0 & 0 & 0 \end{matrix} \right)$ | S11 |
|  |  |

and

|  |  |
| --- | --- |
| $b_{5}\left( n,m\left\vert n^{'},m^{'} \right\vert y \right)=i^{\left\vert m-m^{'} \right\vert-\left\vert m \right\vert-\left\vert m^{'} \right\vert+n^{'}-n+y}\left( -1 \right)^{m-m^{'}}\sqrt{\frac{\left( 2n+1 \right)\left( 2n^{'}+1 \right)}{2n\left( n+1 \right)n^{'}\left( n^{'}+1 \right)}}$  $\sqrt{\left( \left( n+n^{'}+n+y \right)\left( n+n^{'}+1-y \right)\left( y+n-n^{'} \right)(y-n+n^{'})(2y+1) \right)} \times\left( \begin{matrix} n & n^{'} & y \\ m & -m^{'} & -(m-m^{'}) \end{matrix} \right)\left( \begin{matrix} n & n^{'} & y-1 \\ 0 & 0 & 0 \end{matrix} \right)$ | S12 |
|  |  |

where $\left( \begin{matrix} \ldots\\ \ldots\end{matrix} \right)$ denotes the Wigner-3j symbols. More details to the underlying derivation of this operator can be found in the references in the main text. This section defines the operator according to the normalization convention we use.

1. **The fundamental interaction equation**

The fundamental interaction equation on a single particle can be found by applying the superposition principle and the translation operators to write all Mie expansions centered at the observation particle. Inserting equation 3 into equation 2 of the main text gives,

|  |  |
| --- | --- |
| $\sum_{l} c_{l,a}^{exc}\boldsymbol{\Psi}_{l,a}^{\left( 1 \right)}\left( \boldsymbol{r-}\boldsymbol{r}_{a} \right)= \sum_{l^{'}} \left( c_{l^{'}}^{inc}\boldsymbol{\Psi}_{l^{'}}^{\left( 1 \right)}\left( \boldsymbol{r-0} \right)+\sum_{b\neq a}^{I\to\infty} c_{l^{'},b}\boldsymbol{\Psi}_{l^{'}}^{\left( 3 \right)}\left( \boldsymbol{r}-\boldsymbol{r}_{b} \right) \right).$ | S13 |
|  |  |

To express equation S15 as a scalar equation it is necessary to write all fields in a common basis that can then be removed. This is accomplished by applying the spherical vector translation theorem,

|  |  |
| --- | --- |
| $\sum_{l^{'}} c_{l^{'},a}^{exc,}=\sum_{l^{'}l} \left( \mathbb{J}_{l^{'}l}^{a0}\left( \boldsymbol{d}_{a0} \right)c_{l}^{inc}+\sum_{b\neq a}^{I\to\infty} \mathbb{H}_{l^{'}l}^{ab}\left( \boldsymbol{d}_{ab} \right)c_{l,b} \right)$ | S14 |
|  |  |

Note that when dealing with the coefficients, you use the transpose of $\mathbb{H}^{ab^{T}}$, hence we define $\mathbb{H}^{ab}$ as the translation operator for the coefficient equation which is the transpose of the translation operator defined in section 3 of the supplementary information. Multiplying both sides of equation S14 by the scattering operator of the observation particles, $\mathbb{T}_{a},$gives equation 4 in the main manuscript.

1. **Definition of the far field scattered power operator**

The magnetic $(\boldsymbol{\psi}_{far,nm1})$ and electric $(\boldsymbol{\psi}_{far,nm2})$ type far field basis are defined as

|  |  |
| --- | --- |
| $\begin{matrix} \boldsymbol{\psi}_{far,nm1}\left( \hat{\boldsymbol{k}} \right)=\frac{\left( -i \right)^{n}}{\sqrt{2n\left( n+1 \right)}}\left( \nabla Y_{mn}\left( \hat{\boldsymbol{k}} \right)\times\boldsymbol{r} \right) \end{matrix}$  $\begin{matrix} \boldsymbol{\psi}_{far,nm2}\left( \hat{\boldsymbol{k}} \right)=\frac{i\left( -i \right)^{n}}{\sqrt{2n\left( n+1 \right)}}\left( r\nabla Y_{mn}\left( \hat{\boldsymbol{k}} \right) \right) \end{matrix}$, | S15 |
|  |  |

The far field basis can construct the electric and magnetic type spherical vector wave functions from the integral representation

|  |  |
| --- | --- |
| $\boldsymbol{\Psi}_{l}\left( k\boldsymbol{r} \right)=\frac{1}{2\pi}\sum_{q} \int\frac{\partial^{2}\boldsymbol{k}_{\parallel}}{k\left\vert k_{z}^{\pm} \right\vert}\left( \boldsymbol{\psi}_{far,l}\left( \hat{\boldsymbol{k}} \right)\cdot{\hat{\boldsymbol{e}}}_{q}\left( \hat{\boldsymbol{k}} \right) \right)\boldsymbol{\Phi}_{q}\left( \boldsymbol{r};\boldsymbol{k}^{\pm} \right)$, | S16 |
|  |  |

where $\boldsymbol{\psi}_{far,l}\left( \hat{\boldsymbol{k}} \right)\cdot{\hat{\boldsymbol{e}}}_{q}\left( \hat{\boldsymbol{k}} \right)$ is the projection of the far field basis onto the unit vector of the plane wave basis, ${\hat{\boldsymbol{e}}}_{q}$. $\boldsymbol{\Phi}_{q}\left( \boldsymbol{r};\boldsymbol{k}^{\pm} \right)=e^{i\mathbf{k}^{\pm}\cdot\mathbf{r}} {\hat{\boldsymbol{e}}}_{q}(\mathbf{k}^{\pm})$is the plane wave basis function. A plane wave with arbitrary polarization, ${\hat{\boldsymbol{e}}}_{\mathrm{pol}}$, is decomposed to TE and TM orthogonal polarization states such that ${\hat{\boldsymbol{e}}}_{pol}(\boldsymbol{k}^{\pm})=\sum_{q} \left( {\hat{\boldsymbol{e}}}_{pol}(\boldsymbol{k}^{\pm}) \cdot{\hat{\boldsymbol{e}}}_{q}(\boldsymbol{k}^{\pm}) \right){\hat{\boldsymbol{e}}}_{q}(\boldsymbol{k}^{\pm})$, where $q\in[1,2]$. The polarization unit vectors are defined with respect to the outward pointing surface normal at the interface where the incident wave impinges, ${\hat{\boldsymbol{n}}}_{0}=-\hat{\boldsymbol{z}}$. This convention is shown in supplementary figure 1. The first unit vector is in the azimuth direction (TE or s-polarization),

|  |  |
| --- | --- |
| ${\hat{\boldsymbol{e}}}_{1}\left( \boldsymbol{k}^{\pm} \right)=\frac{\hat{\boldsymbol{z}}\times\boldsymbol{k}^{\pm}}{\left\Vert\hat{\boldsymbol{z}}\times\boldsymbol{k}^{\pm} \right\Vert}=\left( -\sin\left( \alpha\right)\hat{\boldsymbol{x}}+\cos\left( \alpha\right)\hat{\boldsymbol{y}}+0\hat{\boldsymbol{z}} \right)=\hat{\boldsymbol{\alpha}} (TE)$ , | S17 |
|  |  |

and the second unit vector in the zenith direction (TM or p-polarization),

|  |  |
| --- | --- |
| ${\hat{\boldsymbol{e}}}_{2}\left( \boldsymbol{k}^{\pm} \right)=\frac{\left( {\hat{\boldsymbol{e}}}_{1}\left( \boldsymbol{k}^{\pm} \right)\times\boldsymbol{k}^{\pm} \right)}{\left\Vert{\hat{\boldsymbol{e}}}_{1}\left( \boldsymbol{k}^{\pm} \right)\times\boldsymbol{k}^{\pm} \right\Vert}=\frac{1}{k}\left( \pm k_{z}\cos\left( \alpha\right)\hat{\boldsymbol{x}}\pm k_{z}\sin\left( \alpha\right)\hat{\boldsymbol{y}}-k_{\parallel}\hat{\boldsymbol{z}} \right)=\hat{\boldsymbol{\beta}} (TM)$ . | S18 |
|  |  |

Defining $\boldsymbol{x}_{lq}={\hat{\boldsymbol{e}}}_{q}\cdot\boldsymbol{\psi}_{far,l}$, then the far field basis for the power flow in the upper and lower hemispheres is $\mathbb{F}_{ll^{'}}^{\pm}= \sum_{q} \iint_{\left| \boldsymbol{k}_{\parallel} \right|\leq k} \frac{d^{2}\boldsymbol{k}_{\parallel}k_{z}^{\pm}}{k\left\| k_{z} \right\|^{2}}\boldsymbol{x}_{lq}^{\dagger}\boldsymbol{x}_{l^{'}q}$.The projections in explicit form are

|  |  |
| --- | --- |
| $\left[ \begin{matrix} \boldsymbol{\psi}_{far,nm1}\left( \hat{\boldsymbol{k}} \right)\cdot{\hat{\boldsymbol{e}}}_{1}\left( \hat{\boldsymbol{k}} \right) & \boldsymbol{\psi}_{far,nm1}\left( \hat{\boldsymbol{k}} \right)\cdot{\hat{\boldsymbol{e}}}_{2}\left( \hat{\boldsymbol{k}} \right) \\ \boldsymbol{\psi}_{far,nm2}\left( \hat{\boldsymbol{k}} \right)\cdot{\hat{\boldsymbol{e}}}_{1}\left( \hat{\boldsymbol{k}} \right) & \boldsymbol{\psi}_{far,nm2}\left( \hat{\boldsymbol{k}} \right)\cdot{\hat{\boldsymbol{e}}}_{2}\left( \hat{\boldsymbol{k}} \right) \end{matrix} \right]=e^{im\alpha}\frac{i\left( -i \right)^{n}}{\sqrt{2n\left( n+1 \right)}}\left[ \begin{matrix} i\tau_{n}^{\left\vert m \right\vert}\left( \beta\right) & m\pi_{n}^{\left\vert m \right\vert}\left( \beta\right) \\ im\pi_{n}^{\left\vert m \right\vert}(\beta) & \tau_{n}^{\left\vert m \right\vert}\left( \beta\right) \end{matrix} \right]$ | S19 |
|  |  |

1. **Derivation of the reflection, transmission, and absorption from a single layer of randomly placed particles with random radii**

Under the scattered field formalism, the total electric field, $\boldsymbol{E}_{tot}$, from this film is a superposition of the scattered field from each particle, $\sum_{a} \boldsymbol{E}_{sca,a}$, and the initial field incident on the particle film, $\boldsymbol{E}_{inc}$. This is written as

| ${\tilde{\boldsymbol{E}}}_{tot}\left( \boldsymbol{r} \right)= \boldsymbol{E}_{inc}\left( \boldsymbol{r} \right)+\sum_{a} {\tilde{\boldsymbol{E}}}_{sca,a}\left( \boldsymbol{r}-{\tilde{\boldsymbol{r}}}_{a} \right)$, | S20 |
| --- | --- |

where $\boldsymbol{r}_{a}\in\mathbb{R}^{3}$ is the geometric center of particle $a$ and the summation is over all (infinitely many) particles. The tilde denotes a random variable. All fields are in frequency domain and the time-harmonic oscillation is implied. In the linear regime, the scattered field of each particle is the result of the particle responding to a driving local field, ${\tilde{\boldsymbol{E}}}_{sca,a}=\mathbb{T}_{a}[{\tilde{\boldsymbol{E}}}_{loc,a}]$. The linear scattering response operator, $\mathbb{T}_{a},$ is a result of the constitutive relations at the particle-host interface and is a property solely of the particle’s shape and material contrast to the host media. The local field experienced by each particle is the superposition of the incident field and the scattered field from all other particles in the film,

| ${\tilde{\boldsymbol{E}}}_{loc,a}\left( {\tilde{\boldsymbol{r}}}_{a} \right)= \boldsymbol{E}_{inc}\left( {\tilde{\boldsymbol{r}}}_{a} \right)+\sum_{b\neq a} {\tilde{\boldsymbol{E}}}_{sca,b}\left( {\tilde{\boldsymbol{r}}}_{a}-{\tilde{\boldsymbol{r}}}_{b} \right)$. | S21 |
| --- | --- |

Equations S23 and S24 as well as the response operator defines a set of coupled linear equations which can be used to solve for the unknown scattered fields given a known incident excitation. Representing fields in terms of a spherical harmonic Mie multipole expansion reduces this coupled equation to a manageable purely algebraic expression. The multipole expansion is

| $\boldsymbol{E}_{inc}\left( \boldsymbol{r} \right)=\boldsymbol{\Psi}^{\left( 1 \right)}\left( \boldsymbol{r} \right)\tilde{c}_{inc}$  $\begin{matrix} {\tilde{\boldsymbol{E}}}_{loc,a}\left( \boldsymbol{r}-{\tilde{\boldsymbol{r}}}_{a} \right)=\boldsymbol{\Psi}^{\left( 1 \right)}\left( \boldsymbol{r}-{\tilde{\boldsymbol{r}}}_{a} \right)\tilde{c}_{a,loc} \\ {\tilde{\boldsymbol{E}}}_{sca,a}\left( \boldsymbol{r}-{\tilde{\boldsymbol{r}}}_{a} \right)=\boldsymbol{\Psi}^{\left( 3 \right)}\left( \boldsymbol{r}-{\tilde{\boldsymbol{r}}}_{a} \right)\tilde{c}_{a}, \end{matrix}$ | S22 |
| --- | --- |

The magnetic field is found through the relation $Z\boldsymbol{H}=\nabla\times\boldsymbol{E}$, where $Z$ is the impedance of the host medium. Under this framework, information about the electrodynamics of the system is expressed through the expansion coefficients of equation S22. Furthermore, the local field coefficients, $\tilde{c}_{a,loc}$, generate the scattering coefficients through the scattering operator, $\mathbb{T}_{a}\in\mathbb{C}^{l\times l}$. Therefore, the system is completely described through the knowledge of the local field experienced by each particle, $\tilde{c}_{a,loc}$, and the particle’s response to that field, $\mathbb{T}_{a}$, dictated by its unique shape and material properties. These equations can be used to derive the fundamental interaction equation governing the system,

| $\tilde{c}_{a}- \mathbb{T}_{a}\sum_{b\neq a} \mathbb{H}^{ab}\left( {\tilde{\boldsymbol{d}}}_{ab} \right)\tilde{c}_{b}=\mathbb{T}_{a}\mathbb{J}^{a0}\left( {\tilde{\boldsymbol{d}}}_{a0} \right)c_{inc},$ | S23 |
| --- | --- |

where $\mathbb{H}^{ab}=\mathbb{J}^{ab}+i\mathbb{Y}^{ab}\in\mathbb{C}^{l\times l}$ is the spherical harmonic translation operator based on the spherical Hankel function and $\mathbb{J}^{ji}$ and $\mathbb{Y}^{ji}$ are the translation operators based on the spherical Bessel functions of the first and second kind, respectively[2].

These operators are a function of the displacement vector between two particles, $\boldsymbol{d}_{ab}$, and enable all fields to be written in a regular multipole expansion centered at particle $a$. In doing this the interaction equation defines a relationship solely between particle coefficients. Furthermore, the interaction equation clearly delineates the contribution of each particle’s scattered field in terms of individual particle properties, $\mathbb{T}_{a}$, the relative spatial location of particles, $\mathbb{H}^{ab}$, and a particle’s location relative to the origin of the external excitation, $\mathbb{J}^{a0}$.

The energy balance relationship for an arbitrary particle, $a$, within the film is[2],

| $W_{ext,a}- W_{abs,a}=W_{sca-i,a}+W_{sca-d,a}$. | S24 |
| --- | --- |

$W_{ext,a}$ is the power removed from the incident field as a result of the presence of particle $a$. $W_{abs,a}$ and $W_{sca-i,a}$ are then the absorbed and scattered power of particle $a$. $W_{sca-d,a}$ is the remaining interference of particle $a$’s scattered field with the scattered field from all other particles[3]. $W_{ext,a}$ and $W_{sca-d,a}$ together define the total interference between particle $a$’s field with the remaining fields in the environment. We explicitly separate these terms because such a distinction becomes meaningful when discussing power balance at the film-level. The $\dagger$ superscript denotes the Hermitian conjugate.

Summing the contributions from each particle then gives the total film-level division of power, $\sum_{a}^{N} \left( W_{ext,a}- W_{abs,a}=W_{sca-i,a}+W_{sca-mix,a} \right)$. It is instructive to note that at the film-level quantiles are in terms of ensemble average values. This result is in fact not an approximation. It comes explicitly from an algebraic repackaging of the particle summation[4]. For films of particles with different shapes, this summation can be partitioned into a sum over all particle shapes followed by the sum of all particles of that shape. In doing this, the film-level energy balance can be recast as an expected energy balance at the particle level,

| $\begin{aligned} ff\int dsw\left( s \right)\left( \left\langle\sigma_{ext,a}\vert s \right\rangle- \left\langle\sigma_{abs,a}\vert s \right\rangle\right) \\ =ff\int dsw\left( s \right)\left( \left\langle\sigma_{sca-i,a}\vert s \right\rangle+\left\langle\sigma_{sca-d,a}\vert s \right\rangle\right). \end{aligned}$ | S25 |
| --- | --- |

Equation S25 has the form of the law of total expectation, where $w\left( s \right)={A_{s}P\left( s \right)}/{\int dsA_{s}P\left( s \right)}$ is the weight factor for integrating over the shape distribution. $\left\langle\sigma_{a}|s \right\rangle=(1/J)\sum_{a}^{J} 2\pi W_{a}/(A_{s}k^{2})$is the summation of the efficiency, $\sigma_{a}=2\pi W_{a}/(A_{s}k^{2})$, of all particles, $a$, that have shape $s$ and cross-sectional area $A_{s}$. The summation is discrete because particles occupy unique positions in space, necessitated by the fact that they cannot physically overlap. The $a$ subscript serves as a reminder that $\sigma_{a}$ is a particle-level efficiency. $W_{a}$ denotes any of the four power flows defined in equation 5. This provides an explicit connection between power flow at the film-level and the expected power balance at the particle-level for films composed of arbitrary convex-shaped inhomogeneities. $\left\langle\sigma|s \right\rangle$ can also be interpreted as the ensemble average over all possible photonic environments surrounding an arbitrary particle of type $s$, given the film is characterized by a joint shape and position distribution. If the shape distribution that cannot satisfy a particular packing fraction, then the joint shape and position distribution will have zero probability. We discuss later how to assign such probabilities for the joint distribution but mention now that an analytic expression for this distribution is an open question in the space packing community[5–7].

In principle, equation 25 has no analytic solution for an arbitrary joint shape and spatial distribution of non-penetrable convex-shaped inhomogeneities. First, the analytic form of such a distribution is generally unknown[5–7]. Second, for a given realization of the joint distribution, each particle’s scattered field is dependent on all scattered fields of the surrounding particles. In an infinitely sized system where Bloch theorem cannot be evoked, this requires a sum of all multiple-scattering paths between (an infinite number of) particles resulting in an infinite Liouville–Neumann series and a hierarchy of conditional distributions[8–10]. Much work has been devoted to truncating this infinite problem. For example, the Twersky approximation of non-self-referencing interactions, quasi-crystallinity, sparse configuration approximations, and pair correlation functions have been widely used[11]. The goal of these works is to simplify the expression of the structure factor and the local field a particle experiences by a priori neglecting perceived negligible terms in the infinite expansion[11]. These ansatzes result in tractable expressions but are valid only in a reduced parameter space. E.g., sparse spatial distributions where particles weakly couple.

Alternatively, we achieve consistent model accuracy under less assumptions by leveraging the growth of modern computer resources and solving the ensemble problem using Monte Carlo integration. The only stipulations to our approach are (1) that the particle film lacks long-range order and (2) that the structure factor is not dependent on the absolute positions of particles. Other than these two stipulations, particles can have any physically realizable convex shape as well as any position distribution. The convexity requirement is the result of the decision to represent fields by a Mie expansion and can, in principle, be relaxed. Under these conditions, it has been shown that the expectation over possible particle positions, $\left\langle\sigma|s \right\rangle$, can be well calculated using Monte Carlo[4]. Each Monte Carlo sample is of a sufficiently large finite sized particle film where the local field of an observation particle deterministically placed at the center of the film is recorded. Therefore, each Monte Carlo sample can be viewed as sampling the density of possible photonic states a particle of shape $s$ would experience if inserted into the film. Our process enables accurate modeling of arbitrary position and particle size distributions. This will be verified in the last section.

Though Mie theory provides a theoretically exact solution for the N-body scattering problem with a well-adopted interpretation of understanding particles as effective atoms, its use in our framework is also motivated by the computational demands of Monte Carlo. Compared to full wave simulations, generalized Mie theory can be hundreds of times faster and require a tenth or less memory.

Returning to equation S25, with the Mie expansion in mind, the total film efficiencies can be expressed in terms of field coefficients as

| $\left\langle\sigma_{ext} \right\rangle\mathcal{=-R}\left\{ \left\langle\left\langle c_{s}^{\dagger}\vert s \right\rangle c_{inc} \right\rangle\right\}$  $\left\langle\sigma_{abs} \right\rangle\mathcal{=-R}\left\{ \left\langle c_{a}^{\dagger}\mathbb{A}_{a}c_{a} \right\rangle\right\}$  $\left\langle\sigma_{sca-i} \right\rangle=\left\langle c_{a}^{\dagger}c_{a} \right\rangle$  $\left\langle\sigma_{sca-d} \right\rangle=\sigma_{sca-d}^{J}+\sigma_{sca-d}^{Y}$  $\left\langle\sigma_{sca-d}^{J} \right\rangle\mathcal{=R}\left\{ \left\langle\sum_{b\neq a} c_{a}^{\dagger}\mathbb{J}^{ab}\left( {\tilde{\boldsymbol{d}}}_{ab} \right)\tilde{c}_{b} \right\rangle\right\}$  $\left\langle\sigma_{sca-d}^{Y} \right\rangle\mathcal{=R}\left\{ i\left\langle\sum_{b\neq a} c_{a}^{\dagger}\mathbb{Y}^{ab}\left( {\tilde{\boldsymbol{d}}}_{ab} \right)\tilde{c}_{b} \right\rangle\right\}$, | S26 |
| --- | --- |

where all outer expectations are a discretized Monte Carlo versions of the expectation of the shape distribution from equation S25. This outer expectation is $\left\langle x \right\rangle=\sum_{s} \gamma_{s}\left\langle x|s \right\rangle$, where $\gamma_{s}=\left( \frac{ff}{2\pi E_{inc}^{2}}\frac{w_{s}}{x_{s}^{2}} \right)$. $E_{inc}$ is the amplitude of the incident field. $x_{s}=ks$ is the size parameter of a particle with size s. $w_{s}$ is a discretization of $w\left( s \right)$. $\mathcal{R}$ denotes the real part. The inner expectations define the particle-level efficiencies in terms of scattering coefficients. $\mathbb{A}_{a}=-\mathcal{R}\left( \mathbb{T}_{a}^{-1}+1 \right)\in\mathbb{C}^{l\times l}$ is the absorption operator for particle $a$[2].

The total reflection in the far field from the film is then

| $R_{sca}=-\mathcal{R}\left\{ \begin{aligned} \left\langle c_{a}^{\dagger}\mathbb{F}^{-}c_{a} \right\rangle\\ +\left\langle\sum_{b\neq a} c_{a}^{\dagger}\mathbb{F}^{-}\mathbb{J}^{ab}c_{b} \right\rangle\end{aligned} \right\}$ | S27 |
| --- | --- |

where $\mathbb{F}^{\pm}\in\mathbb{C}^{l\times l}$ is the far field scattered power operator in matrix form. The superscript denotes the transmission ($+$) and reflection ($-$) region, defined such that $k_{z}^{\pm}=\pm\left( k^{2}-k_{\parallel}^{2} \right)^{1/2}$ is positive in transmission and negative in reflection. Each element of the far field scattered power matrix is defined as

| $\mathbb{F}_{ll^{'}}^{\pm}= \sum_{q} \iint_{\left\vert\boldsymbol{k}_{\parallel} \right\vert\leq k} \frac{d^{2}\boldsymbol{k}_{\parallel}k_{z}^{\pm}}{k\left\Vert k_{z} \right\Vert^{2}}\boldsymbol{x}_{lq}^{\dagger}\boldsymbol{x}_{l^{'}q},$ | S28 |
| --- | --- |

where $\boldsymbol{x}_{lq}\boldsymbol{=}{\hat{\boldsymbol{e}}}_{q}\cdot\boldsymbol{\psi}_{far,l}$ is the projection of the far-field Mie basis, $\boldsymbol{\psi}_{far,l}=\lim_{r\to\infty} \boldsymbol{\Psi}_{l}^{\left( 3 \right)}$, onto the two orthogonal unit vectors, ${\hat{\boldsymbol{e}}}_{q}$, defining the integration plane. $q\in\left\{ TE=0, TM=1 \right\}$, where TE and TM denote transverse electric and magnetic, respectively, with respect to the integration plane. The surface integral is over the component of the wavevector parallel to the integration plane, $\boldsymbol{k}_{\boldsymbol{\parallel}}$, and only propagating wavevectors, $k_{\boldsymbol{\parallel}}\boldsymbol{\leq}k$, are considered in the far field. $\left\langle c_{a}^{\dagger}\mathbb{F}^{\pm}c_{a}|s \right\rangle$ is the independent and $\left\langle\sum_{b\neq a} c_{a}^{\dagger}\mathbb{F}^{\pm}\mathbb{J}^{ab}c_{b}|s \right\rangle$ is the dependent scattering contribution between particles in each hemisphere. The total film transmission, reflection, and absorption is

| $T=1-R_{sca}-A,$  $R=R_{sca}$  $A=\sigma_{abs},$ | S29 |
| --- | --- |

This includes not just the contribution from both the coherent but also the contribution of the incoherent field.

It is worth mentioning that the Monte Carlo generalized Mie method derived in equations S20-S29 can simulate random films with any particle shapes subject to the condition that a plane can be defined between any two particles. Therefore, though we state that this method is valid for convex shaped particles, the more rigorous condition is in fact looser and concave particles can be simulated if the particles do not intertwine. In general, care must be taken regarding near-field coupling using the generalized Mie expansion if two particles have overlapping circumscribing radii. In this case, given a separating plane can be defined, the translation operator in equation S23 is generalized to an operator of three parts: (1) a Mie harmonic to plane-wave transform at the separating plane, then (2) translation, followed by (3) a plane-wave to Mie harmonic transform. The convergence criteria of this operator is a function of the appreciable multipoles interacting in the near field[12,13]. Clearly, this can require care in determining the appropriate multipole cutoff to fully represent the scattering response. Since it is not necessary to introduce such complexity to demonstrate a filtering effect, we constrain our study to spheres of different radii distributions. This also simplifies the expression for $\mathbb{T}_{a}$, which is then the well-known and fast to calculate Mie solution of a sphere. Therefore, in the remaining sections of this manuscript, the shape factor will be synonymous with particle radii.

1. **Derivation of the reflection, transmission, and absorption from a single layer of randomly placed particles with random radii without hemispherical integrations**

By virtue of power conservation, the incident intensity that is absorbed $(Abs)$ and scattered $(Sca$) by an infinite particle film is found by summing the interaction of each particle in the film

|  |  |
| --- | --- |
| $Abs=\frac{C_{abs}}{A} = \lim_{N\to\infty}\frac{ff}{\sum_{j=1}^{N} A_{j}}\sum_{j=1}^{N} C_{abs,j}$ | S30 |
|  |  |

and

|  |  |
| --- | --- |
| $Sca=\frac{C_{sca}}{A} = \lim_{N\to\infty}\frac{ff}{\sum_{j=1}^{N} A_{j}}\sum_{j=1}^{N} C_{sca-i,j}+C_{sca-d,j},$ | S31 |
|  |  |

where $A$ is the total film area (infinite), $ff=\sum_{j=1}^{N} A_{j}/A$ is the total particle area fill fraction, $A_{j}=\pi r_{j}^{2}$ is particle $j$’s cross-sectional area, $r_{j}$ is particle $j$’s radii, and $N$ is the number of particles in the film (infinite). The film’s total absorption cross section is the sum of the absorption cross section of every particle, $C_{abs}= \lim_{N\to\infty} \sum_{j=1}^{N} C_{abs,j}$, where $C_{abs,j}$ is the absorption cross section of particle $j$. The particle level scattering cross section, ${C_{sca,j}=C}_{sca-i,j}+C_{sca-d,j}$, is the sum of the scattered field from particle $j$ arising from the interaction with the incident field, $C_{sca-i,j}$, and the interaction with the multiply scattered field, $C_{sca-d,j}$. The total scattering cross section is then found by summing over all particles, $C_{sca}= \lim_{N\to\infty} \sum_{j=1}^{N} C_{sca,j}$. From the definition of particle efficiency, we can write $C_{j}=A_{j}\sigma_{j}$ for particle $j$. In doing this, we find the last equality in equations S30 and S31 represent a weighted average,

|  |  |
| --- | --- |
| $Abs= ff\lim_{N\to\infty}\sum_{j=1}^{N} \frac{A_{j}}{\sum_{j=1}^{N} A_{j}}\sigma_{abs,j}$ | S32 |
|  |  |

and

|  |  |
| --- | --- |
| $Sca= ff\lim_{N\to\infty}\sum_{j=1}^{N} \frac{A_{j}}{\sum_{j=1}^{N} A_{j}}\left( \sigma_{sca-i,j}+\sigma_{sca-d,j} \right).$ | S33 |
|  |  |

Since multiple particles can have the same cross-sectional area, we can factor common $A_{j}$ and break $N$ into $K\leq N$ disjoint sets. Then $K$ is the number of unique particle radii, $r_{k}$, and $n_{k}$ is the number of particles that have radii $r_{k}$. Therefore, $N=\sum_{k=1}^{K} n_{k}$ and $\sum_{j=1}^{N} A_{j}= \sum_{k=1}^{K} n_{k}A_{k}$. The total efficiency (absorption or scattered) coming from all particles of radius $r_{k}$ is $\sigma_{k}= \sum_{s=1}^{n_{k}} \sigma_{s}$. It is important to note that even though all $s$ particles have the same cross-sectional area; their efficiency is unique due to each particle experiencing potentially different coupling from neighboring particles. Factoring out common radii in equations S32 and S33 gives

|  |  |
| --- | --- |
| $Abs= ff\lim_{K\to\infty}\sum_{k=1}^{K} \frac{n_{k}A_{k}}{\sum_{k=1}^{K} n_{k}A_{k}}\left( \lim_{n_{k}\to\infty}\frac{1}{n_{k}}\sum_{s=1}^{n_{k}} \sigma_{abs,s} \right)=ff \left\langle\sigma_{abs} \right\rangle$ | S34 |
|  |  |

and

|  |  |
| --- | --- |
| $Sca=ff\lim_{K\to\infty}\sum_{k=1}^{K} \frac{n_{k}A_{k}}{\sum_{k=1}^{K} n_{k}A_{k}}\left( \lim_{n_{k}\to\infty}\frac{1}{n_{k}}\sum_{s=1}^{n_{k}} \sigma_{sca-i,s}+\sigma_{sca-d,s} \right)= ff \left\langle\sigma_{sca} \right\rangle.$ | S35 |
|  |  |

Equations S34 and S35 are in the form of total expectation. The weight $w_{k}={n_{k}A_{k}}/{\sum_{k=1}^{K} n_{k}A_{k}}$ represents a realization of the probability distribution describing the proportion of area particles of a distinct radius contributes to the total particle area in the film, where $n_{k}A_{k}\geq0$ and $\sum_{k=1}^{K} w_{k}=1$. This distribution is denoted as $P(s)$ in the main text, where a discrete realization of the distribution would be $w=\{w_{1},w_{2},\ldots, w_{K}\}$. The scattered field in the forward and backward hemisphere can be approximated by

|  |  |
| --- | --- |
| $Sca_{forward}\approx ff\lim_{K\to\infty}\sum_{k=1}^{K} \frac{n_{k}A_{k}}{\sum_{k=1}^{K} n_{k}A_{k}}\left( \lim_{n_{k}\to\infty}\frac{1}{n_{k}}\sum_{s=1}^{n_{k}} \sigma_{sca,s}\left( \theta=0^{\circ} \right) \right)=ff\left\langle\sigma_{sca}\left( \theta=0^{\circ} \right) \right\rangle$ | S36 |
|  |  |

and

|  |  |
| --- | --- |
| $Sca_{backward}\approx ff\lim_{K\to\infty}\sum_{k=1}^{K} \frac{n_{k}A_{k}}{\sum_{k=1}^{K} n_{k}A_{k}}\left( \lim_{n_{k}\to\infty}\frac{1}{n_{k}}\sum_{s=1}^{n_{k}} \sigma_{sca,s}\left( \theta=180^{\circ} \right) \right)=ff\left\langle\sigma_{sca}\left( \theta=180^{\circ} \right) \right\rangle$ | S37 |
|  |  |

where $\sigma_{sca,i}\left( \theta\right)$ = $\sigma_{sca-i,i}(\theta)+\sigma_{sca-d,i}(\theta)$ is the angle resolved scattering efficiency of particle $i$. For an infinite particle film of monolayer thickness, we write the absorption as $A=ff\left\langle\sigma_{abs} \right\rangle$. The fraction of photons elastically scattered in an infinite particle film of monolayer thickness is written as $Sca=ff\left\langle\sigma_{sca} \right\rangle\approx{Sca}_{backward}+Sca_{forward}$. The reflection from the infinite particle film is then defined as $R\approx Sca_{backward}\approx ff\left\langle\sigma_{sca,i} \right\rangle/(1+FBR_{A}$), by direct substitution of $Sca\approx\left( 1+FBR_{A} \right)Sca_{backward}$. The transmission is found through energy conservation $T+R+A\approx1$.

1. **Comparison of absorption and scattering spectra to single scattering properties.**

Figures 3 and 5 in the main text highlight the role individual scattering characters of particles plays on filter design. This section expands on the analysis of the main text in this regard. Figures S1 - S4 plot the absorption and scattering profile of isolated particles and compares this to particles embedded in a random film, where the size distributions and correlation function is the same as the main text. The goal of this comparison is to see how random coupling alters the overall scattering and absorption efficiency of the particles within the film.

The plots are both wavelength and particle size resolved and the color bar is normalized so that summing over all unique particle radii will return the total absorption and scattering efficiency of the film, as dictated by equation S32 and S33. To conceptually understand this normalization, recall that the total scattering and absorption efficiency of the film is the expected value of the efficiencies of the particles within the film. By the law of total expectation, the expected efficiency can be written generically as $\left\langle\sigma\right\rangle=\sum\sigma(s)P(s)$, where $s$ is the unique particle shape, $\sigma(s)$ is the expected efficiency for that particle shape (averaged over all positions), and $P(s)$ is the probability of observing shape $s$. The color bar is $\sigma(s)P(s)$.

Figures S1 - S4 shows that a primary role of particle coupling is to smooth/blur the scattering resonance peaks of isolated particles in order to flatten and broaden the passband/stopbands. For example, in the shortpass filter, particle coupling smooths takes the isolated scattering peak near 7.3 um and smooths it across the 7 – 9 um spectral window. This creates the reliable and relatively flat stopband from 7.3 – 9 um seen in the figure 2 of the main text. The results of figure S1 further support the idea that the optimizer uses particle coupling as a tool to broaden/smooth otherwise narrowband isolated scattering resonances across the spectral regions of interest.


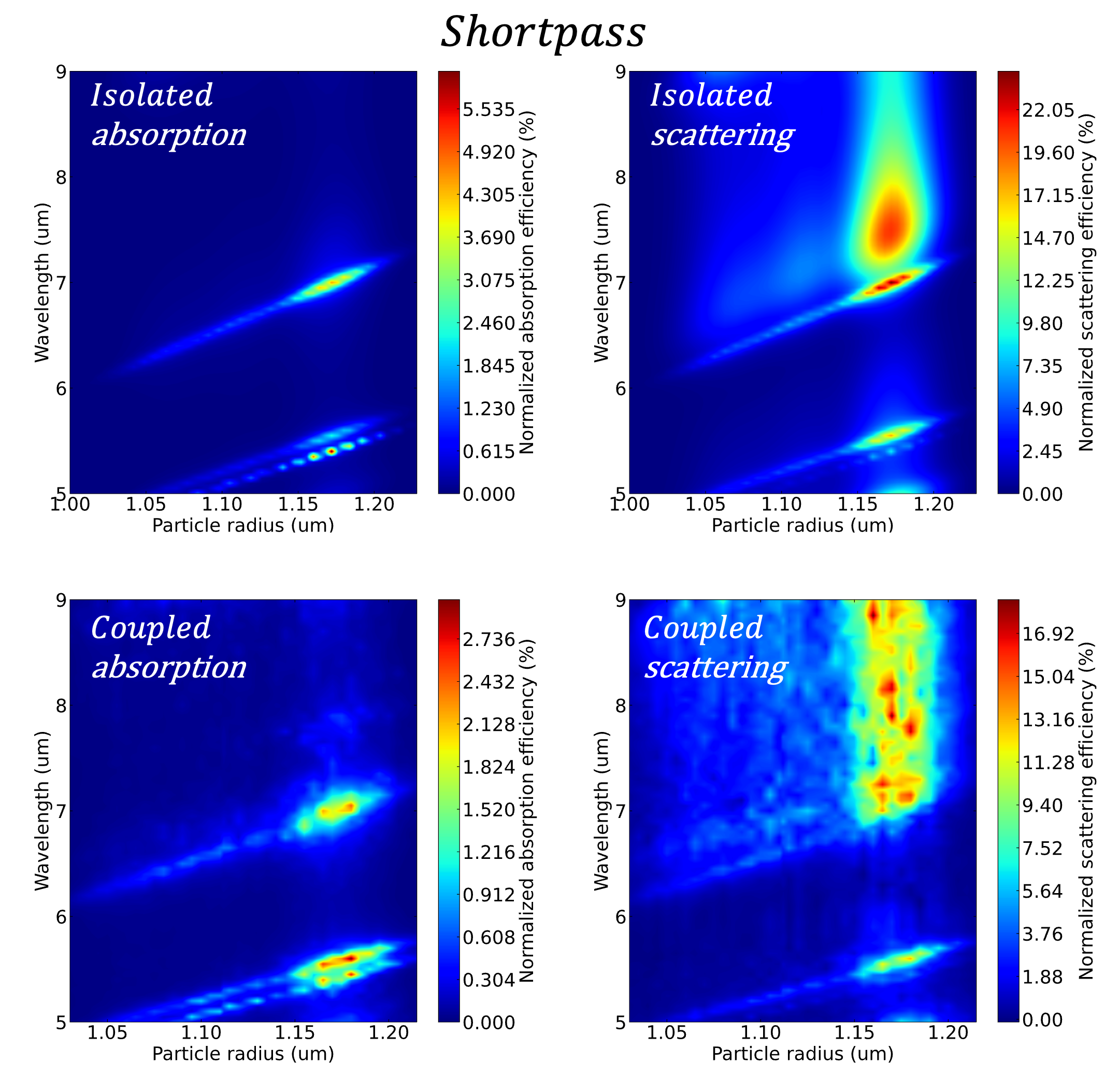


**Figure S1.** Contribution of a random film’s total absorption (left column) and scattering (right column) efficiency as a function of the radius of particles within the film. The top row is calculated using uncoupled particles. Therefore, the sum of all particles can produce a total (film level) scattering and absorption efficiency that is not physical. Nonetheless, this row provides insight into the scattering and absorption behavior of the embedded particles when in a homogeneous environment. The bottom row gives the values when particles are coupled. This the sum of all particles efficiencies for this row does define a proper power balance relation.


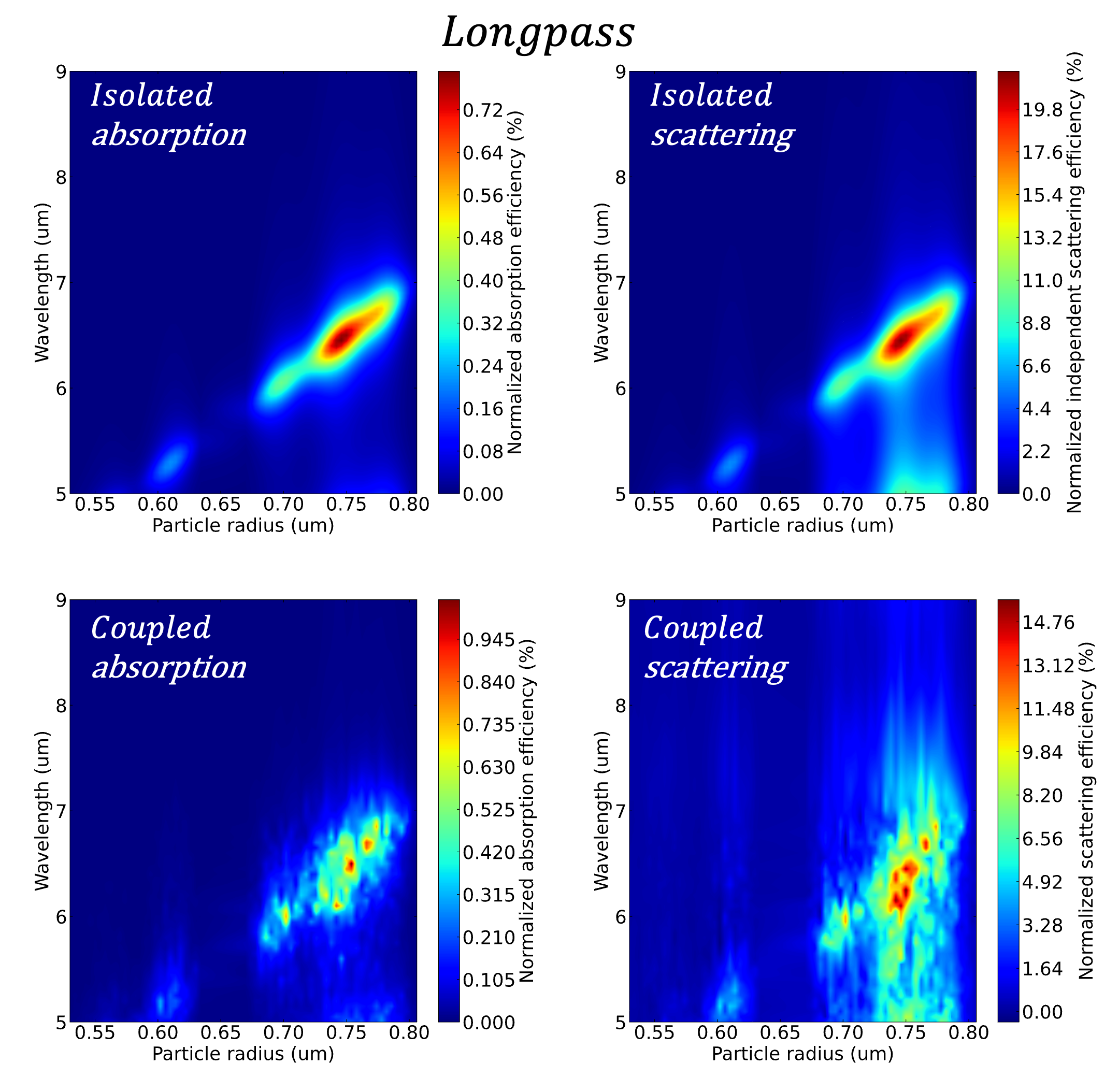


**Figure S2.** Contribution of a random film’s total absorption (left column) and scattering (right column) efficiency as a function of the radius of particles within the film. The top row is calculated using uncoupled particles. Therefore, the sum of all particles can produce a total (film level) scattering and absorption efficiency that is not physical. Nonetheless, this row provides insight into the scattering and absorption behavior of the embedded particles when in a homogeneous environment. The bottom row gives the values when particles are coupled. This the sum of all particles efficiencies for this row does define a proper power balance relation.


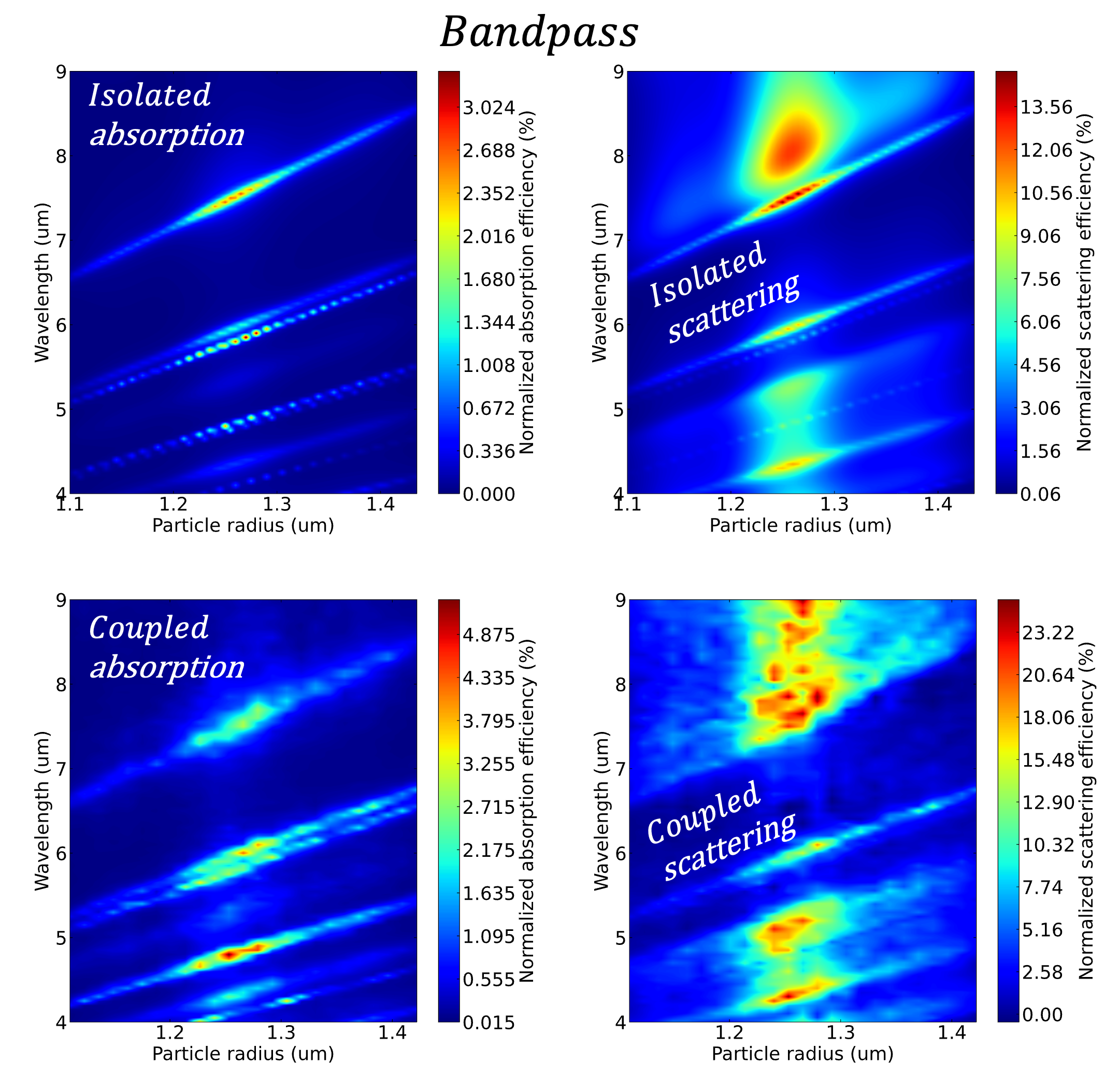


**Figure S3.** Contribution of a random film’s total absorption (left column) and scattering (right column) efficiency as a function of the radius of particles within the film. The top row is calculated using uncoupled particles. Therefore, the sum of all particles can produce a total (film level) scattering and absorption efficiency that is not physical. Nonetheless, this row provides insight into the scattering and absorption behavior of the embedded particles when in a homogeneous environment. The bottom row gives the values when particles are coupled. This the sum of all particles efficiencies for this row does define a proper power balance relation.


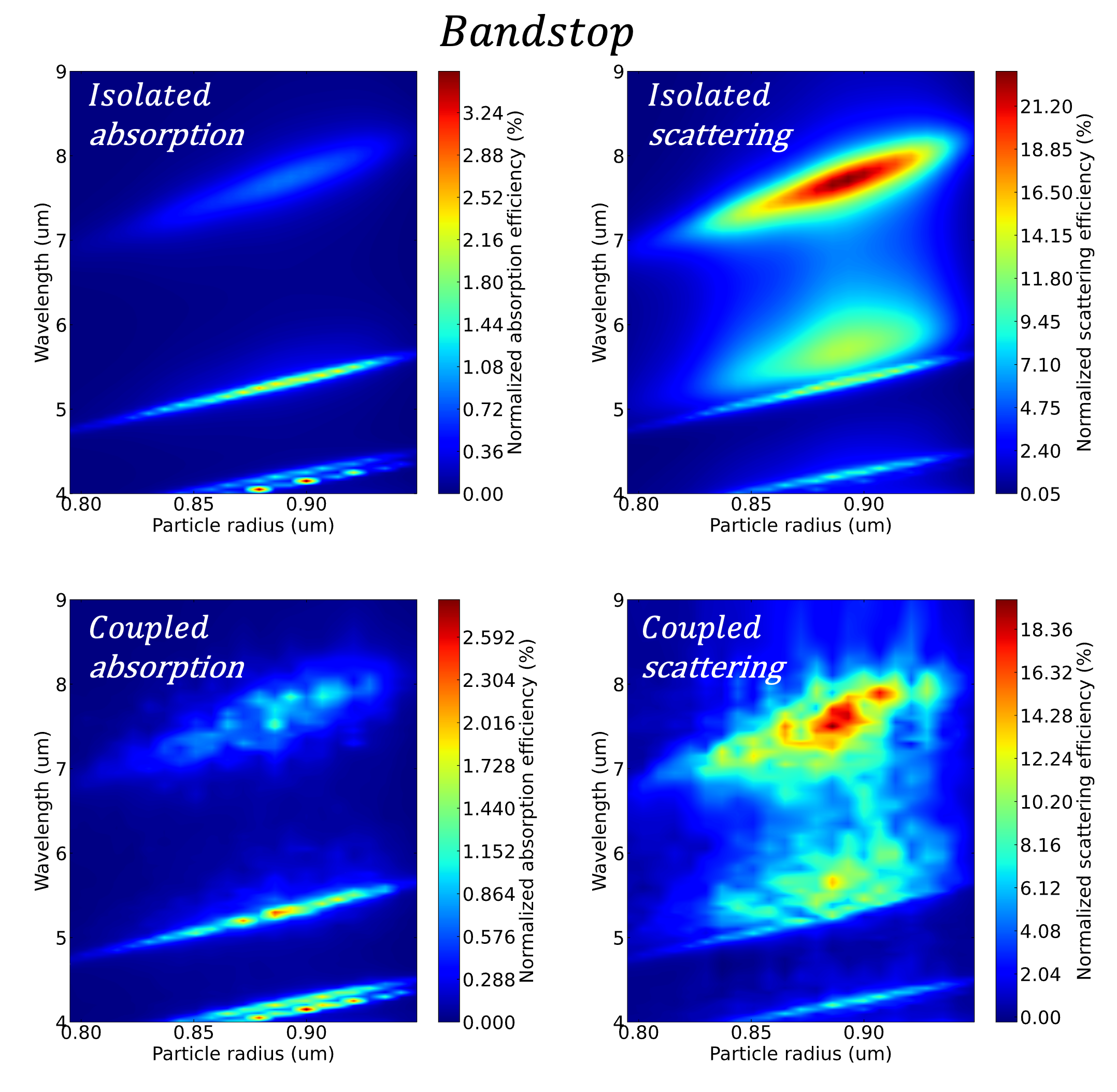


**Figure S4.** Contribution of a random film’s total absorption (left column) and scattering (right column) efficiency as a function of the radius of particles within the film. The top row is calculated using uncoupled particles. Therefore, the sum of all particles can produce a total (film level) scattering and absorption efficiency that is not physical. Nonetheless, this row provides insight into the scattering and absorption behavior of the embedded particles when in a homogeneous environment. The bottom row gives the values when particles are coupled. This the sum of all particles efficiencies for this row does define a proper power balance relation.

1. **Comparison of reflection, transmission, and absorption spectra to quasistatic effective medium theories.**

Given the computational complexity of the method outlined in sections 1 – 7, it is prudent to discuss how this method compares to simpler models. Figure S5 compares the spectra of the random film’s generated in the main text to their Maxwell-Garnett and Bruggeman counterparts. Thin film transmission matrix calculations are used to calculate the spectra of all effective media simulations. In this case, the film thickness for each effective medium calculation was defined by the mode diameter of the particle radii distribution shown in figure 2. In all cases the mode (most likely) particle diameter is close to both the maximum and mean particle diameter. Changing the thickness between these three parameters did not have a significant effect on the resulting spectra. Filling fractions are chosen to be the same as the optimized filling fraction shown in figure 2 of the main text. It is clear from figure S5 that the Maxwell-Garnett and Bruggeman effective media do not accurately predict the true spectra of the particle filters. I.e., these models do not match the full-wave finite-difference time-domain results or the Monte Carlo generalized Mie results. Furthermore, by comparison, the effective media predict much lower quality filters. In multiple cases the reflection and transmission spectra is predominantly flat throughout the spectral range. In these cases, there is no transition from a dominant reflection region to a dominant transmission region at all.


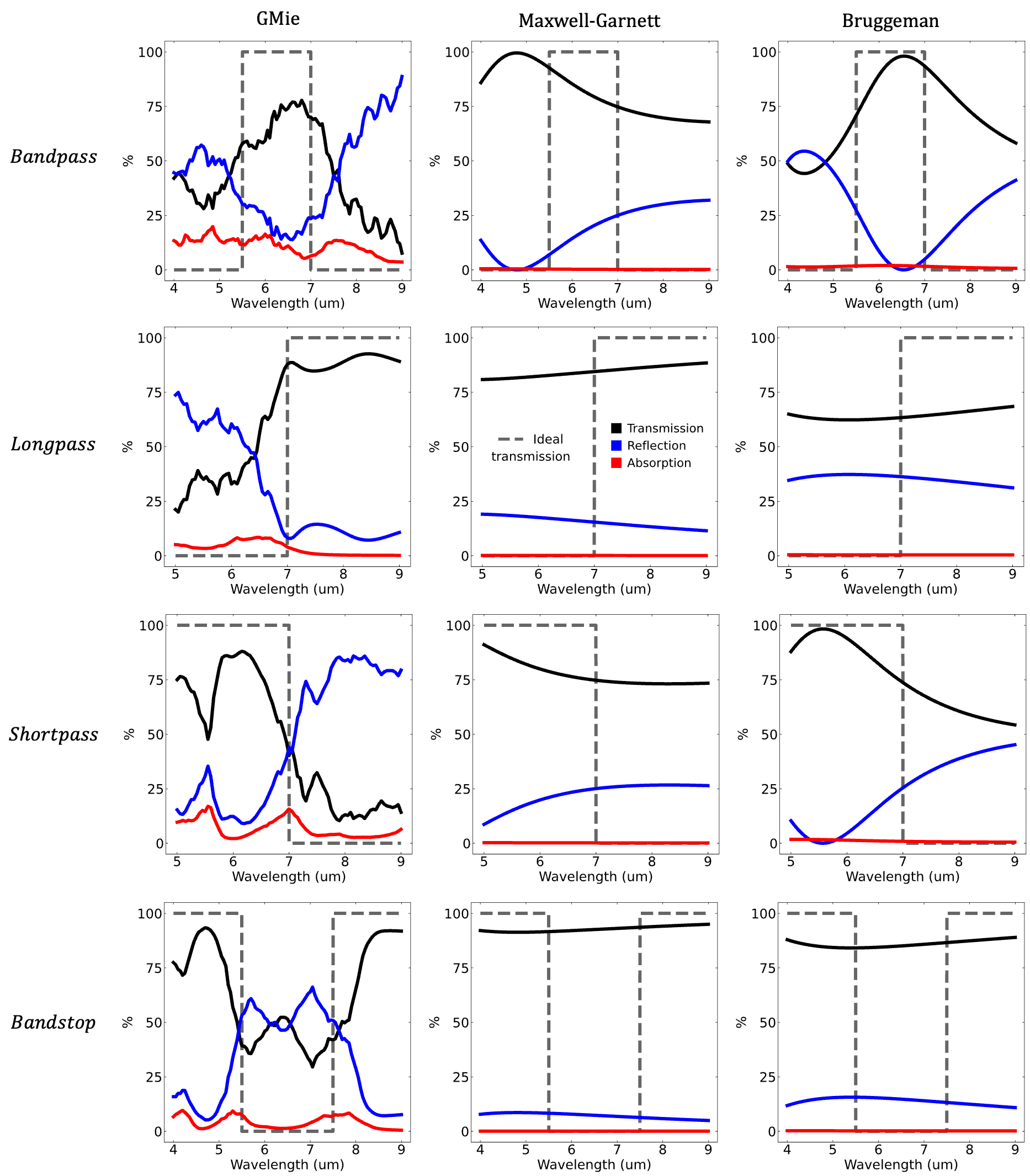


**Figure S5.** Comparison of particle filters made from randomly distributed Mie resonant particles confined to a single layer and filters made from analogous effective media based on the Maxwell-Garnett and Bruggeman approximations. The thickness and filling fraction of the effective medium models are determined by the optimizer distribution given in figure 2. The input parameters are: bandpass (thickness = 1256 nm, filling fraction = 56%), longpass (thickness = 745 nm, filling fraction = 41%), shortpass (thickness = 1170 nm, filling fraction = 50%), bandstop (thickness = 890 nm, filling fraction = 26%).

1. **Comparison of reflection, transmission, and absorption spectra between random films and dense periodic arrays.**

Given that the spectral characteristics of the random films are dependent on the Mie scattering characteristics of the individual particles, it is instructive to explore the differences between disorder (in both particle radii and position) and ordered in shaping the film’s spectra. It should be noted that the use of periodic arrays for designing spectral filters is well known and filters can be made of exceptional quality using this method [14–16]. The benefits and tradeoffs of introducing disorder then is application dependent, as discussed in the introduction. The goal of this section is to present a periodic array that is an ordered analogy to the random film systems, to develop intuition by comparison.

Figure S6 compares the disorder-driven films from the main text to an analogous ordered and periodic array of particles. The square array is periodically repeated using Bloch boundary conditions, with particle sizes given by the mode of the particle distribution given in figure 2. The most likely particle size is used as this is the particle size that is the primarily contributor to the filter response. Figure S6 shows that disorder both in the position and radii distribution has a smoothing effect of spectral features. For example, the passband of the longpass filter is flattened and broadened in the spectral range as a result of increasing disorder. In the ordered array, resonances in the reflection spectra are enhanced as a result of collective constructive interference. In multiple cases this has a deleterious effect on the filter quality. For example, in the passband of the shortpass filter. Overall, the spectral match between the ordered and disordered system, as well as filter quality, is substantially better compared to effective medium approximations discussed in section 9. This is because the ordered system properly accounts for individual Mie resonances. The particle-particle coupling effects are also properly modeled. The overall conclusion is that ordered arrays produce more heightened and narrow band spectral features. Disorder smooths this density of states across the spectra. In some cases, this can be substantial.


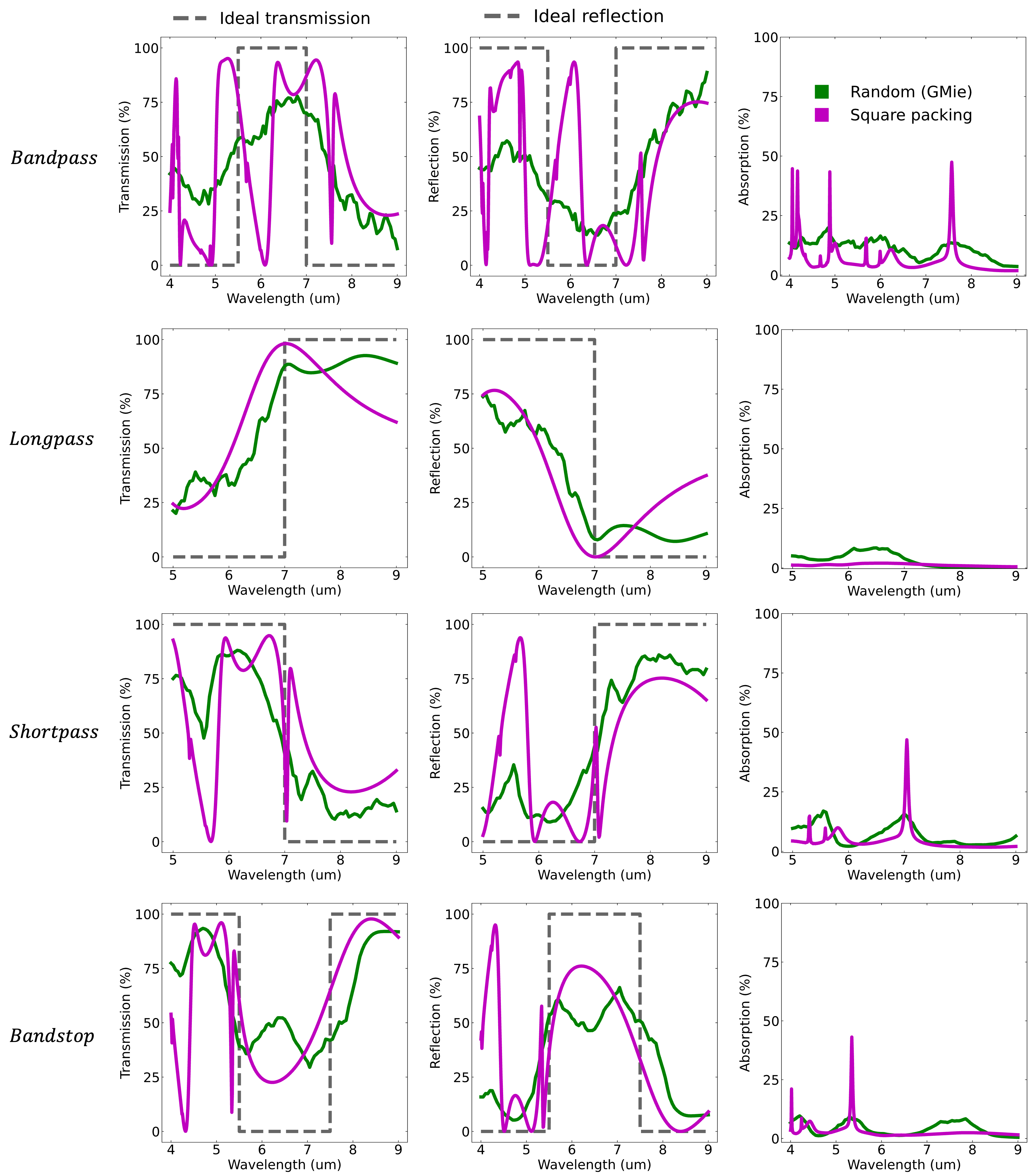


**Figure S6.** Comparison of particle filters made from randomly distributed Mie resonant particles confined to a single layer and filters made from a dense periodic square packing of particles. Particle diameters are 1256 nm (bandpass), 745 nm (longpass), 1170 nm (shortpass), and 890 nm (bandstop). Solid green lines are the spectra of the random films, as shown in the main manuscript. Solid purple lines are calculated are the spectra from a periodic square dense packing of particles. The dashed grey lines give the ideal reflection and transmission spectra.

1. **Additional supplementary figures**


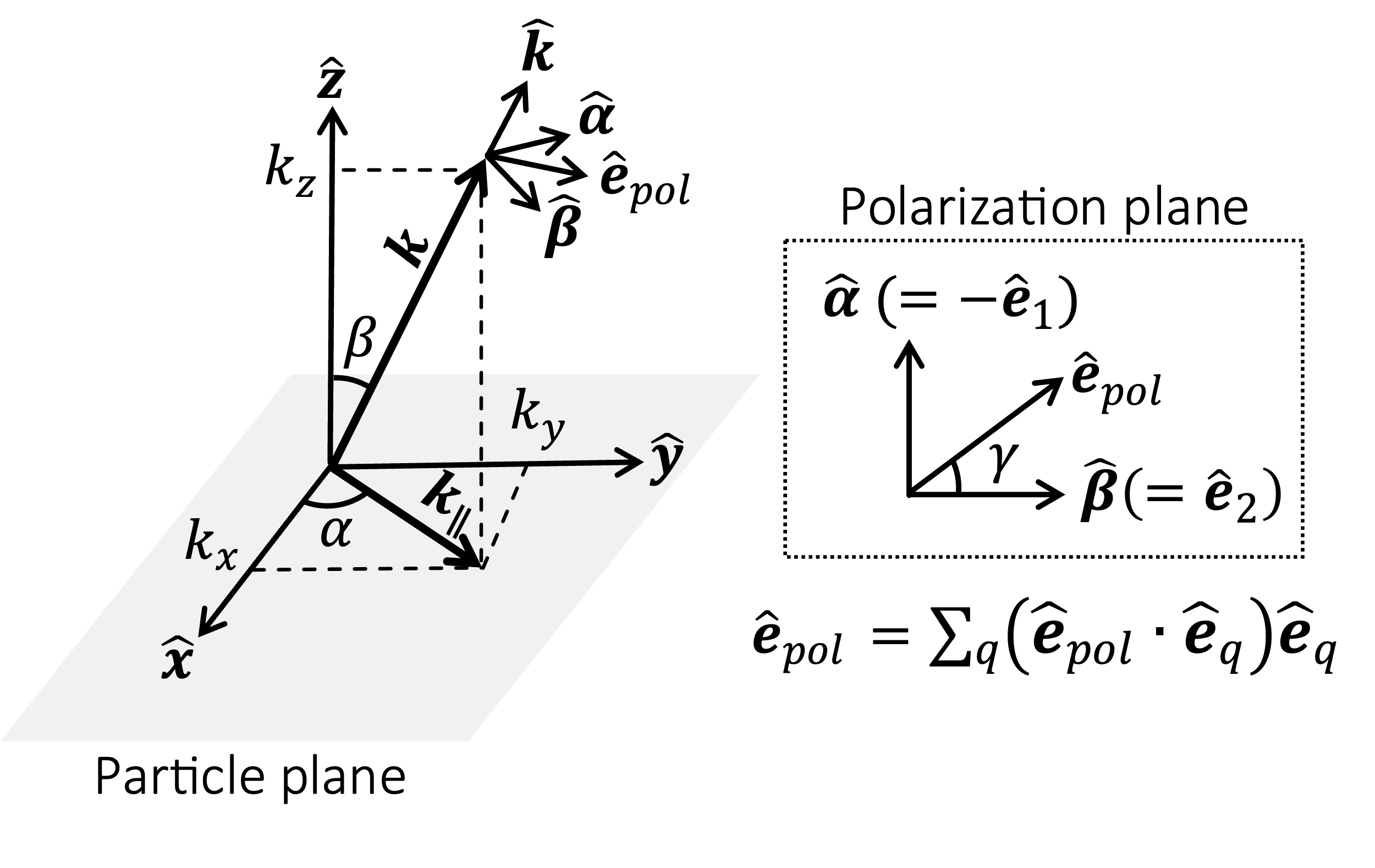


**Figure S7.** Schematic of the coordinate system used for all spatial variables.


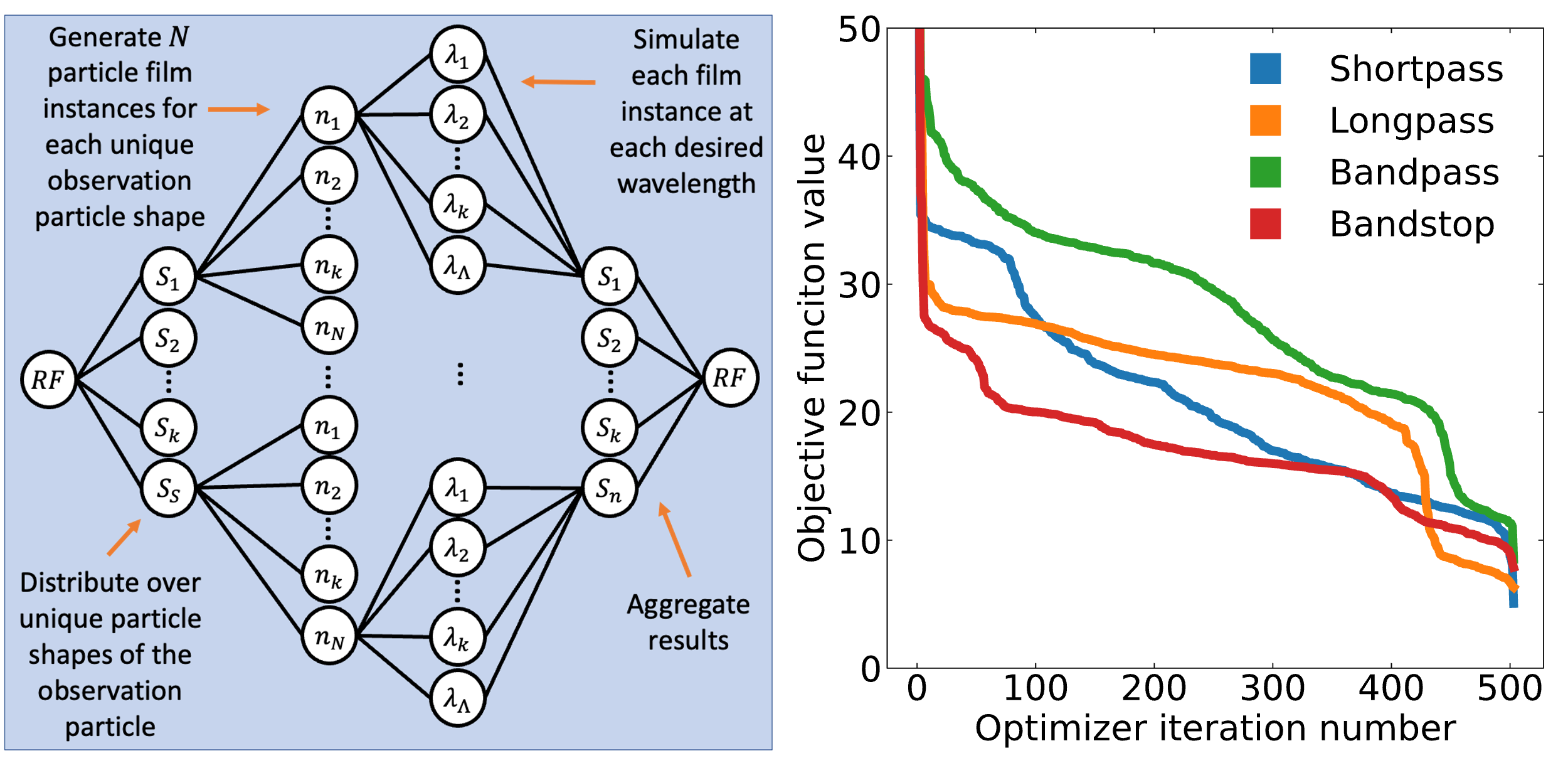


**Figure S8.** (Left) Schematic of the distributed network used to simulate a single random film. The random film (RF) is characterized by a discretized size distribution and area fill fraction. The program first distributes the calculation for each unique particle size (S) and, for each of those, distributes the task of generating unique spatial distribution samples (n). Each spatial distribution then distributes the task of solving the electromagnetic problem of that sample for each desired wavelength. The results are then compiled. Seed particle distributions and fill fractions are first simulated to construct a prior. From this, the optimal solution to the expected improvement function picks the next proposed sample point. Each sample is a discrete probability distribution of particle sizes and an area fill fraction. The sample is then simulated and the reflection and transmission result is stored in a global database. The optimizer then compiles all data in the global database and uses the totality of the data to update the prior. The process then iterates by again choosing the next sample point through the acquisition function on the updated prior. (Right) Best (minimum) objective function for the longpass (orange), shortpass (blue), bandpass (green), and bandstop (red) filter as a function of Bayesian optimizer iterations.


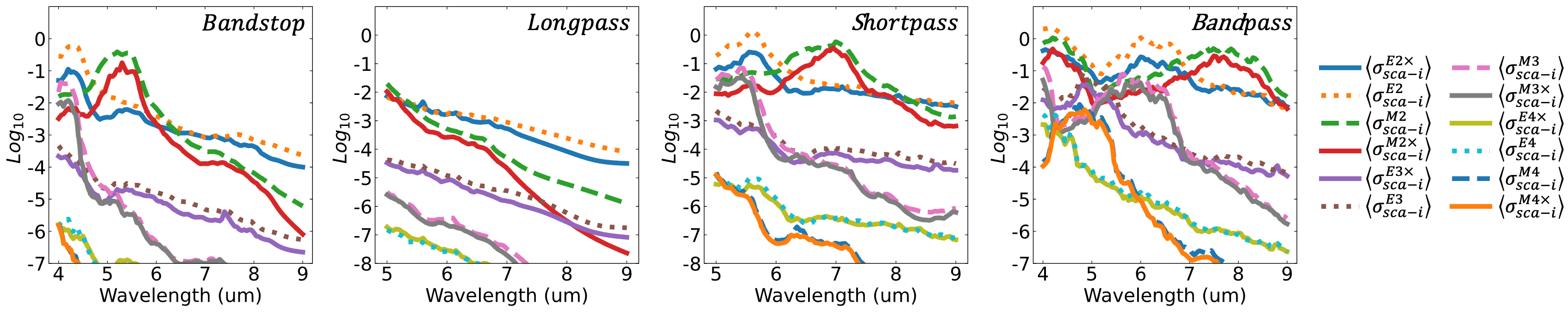


**Figure S9.** The film’s independent scattering cross section decomposed into the Mie modes beyond the electric and magnetic dipole harmonics ($n\in[2,3,4]$). Like figure 5 in the main text, for each quantum polar number, the independent scattering efficiency is the sum of the corresponding azimuthal polar numbers. Parity is written with respect to the polarization (and cross-polarization) of the incident plane wave. According to the null points of the electric-type modes, $n=1$ corresponds to dipole, $n=2$ is quadrupole, $n=3$ is hexapole, and $n=4$ is octupole radiation. The E and H superscript denote electric and magnetic-type harmonics, respectively.


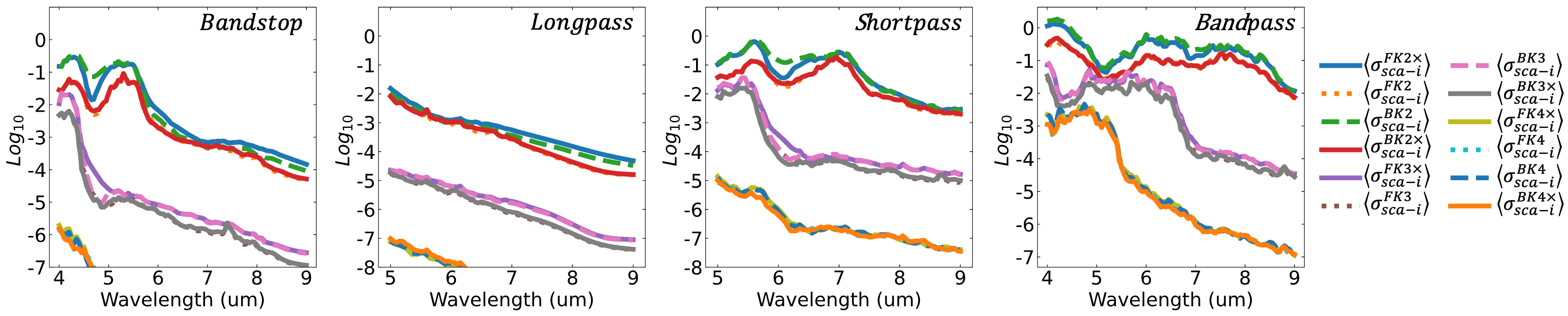


**Figure S4.** The film’s independent scattering cross section decomposed into the Kerker modes beyond the forward and backward first-order harmonics ($n\in[2,3,4]$). Like figure 5 in the main text, for each quantum polar number, the independent scattering efficiency is the sum of the corresponding azimuthal polar numbers. Parity is written with respect to the polarization (and cross-polarization) of the incident plane wave. The F and B superscript denote forward and backward-type harmonics, respectively.

**Supplementary Information References**

1. C. F. Bohren and D. R. Huffman, *Absorption and Scattering of Light by Small Particles* (Wiley-VCH, 2004).

2. D. Mackowski, "The Extension of Mie Theory to Multiple Spheres," in *The Mie Theory: Basics and Applications*, W. Hergert and T. Wriedt, eds., Springer Series in Optical Sciences (Springer, 2012), pp. 223–256.

3. D. W. Mackowski, "Calculation of total cross sections of multiple-sphere clusters," J. Opt. Soc. Am. A **11**(11), 2851 (1994).

4. P. R. Wray and H. A. Atwater, "Light–Matter Interactions in Films of Randomly Distributed Unidirectionally Scattering Dielectric Nanoparticles," ACS Photonics **7**(8), 2105–2114 (2020).

5. D. de Laat, F. M. de O. Filho, and F. Vallentin, "Upper bounds for packings of spheres of several radii," Forum math. Sigma **2**, e23 (2014).

6. C. N. Likos and C. L. Henley, "Complex alloy phases for binary hard-disc mixtures," Philosophical Magazine B **68**(1), 85–113 (1993).

7. N. Bédaride and T. Fernique, "Density of Binary Disc Packings: The 9 Compact Packings," (2021).

8. M. I. Mishchenko, J. M. Dlugach, M. A. Yurkin, L. Bi, B. Cairns, L. Liu, R. L. Panetta, L. D. Travis, P. Yang, and N. T. Zakharova, "First-principles modeling of electromagnetic scattering by discrete and discretely heterogeneous random media," Physics Reports **632**, 1–75 (2016).

9. P. C. Waterman and R. Truell, "Multiple Scattering of Waves," 27 (n.d.).

10. M. I. Mishchenko, "Multiple scattering, radiative transfer, and weak localization in discrete random media: Unified microphysical approach," Reviews of Geophysics **46**(2), (2008).

11. T. C. Choy, *Effective Medium Theory: Principles and Applications*, International Series of Monographs on Physics (Clarendon Press, 1999).

12. A. Egel, D. Theobald, Y. Donie, U. Lemmer, and G. Gomard, "Light scattering by oblate particles near planar interfaces: on the validity of the T-matrix approach," Opt. Express **24**(22), 25154 (2016).

13. A. Egel, S. W. Kettlitz, and U. Lemmer, "Efficient evaluation of Sommerfeld integrals for the optical simulation of many scattering particles in planarly layered media," J. Opt. Soc. Am. A **33**(4), 698 (2016).

14. J. D. Joannopoulos, ed., *Photonic Crystals: Molding the Flow of Light*, 2nd ed (Princeton University Press, 2008).

15. A. A. Miskevich and V. A. Loiko, "Layered periodic disperse structures of spherical alumina particles: Coherent transmittance and reflectance spectra," Journal of Quantitative Spectroscopy and Radiative Transfer **136**, 58–70 (2014).

16. M. Song, D. Wang, S. Peana, S. Choudhury, P. Nyga, Z. A. Kudyshev, H. Yu, A. Boltasseva, V. M. Shalaev, and A. V. Kildishev, "Colors with plasmonic nanostructures: A full-spectrum review," Applied Physics Reviews **6**(4), 041308 (2019).
